# A systematic-search-and-review of registered pharmacological therapies investigated to improve neuro-recovery after a stroke

**DOI:** 10.3389/fneur.2024.1346177

**Published:** 2024-01-31

**Authors:** Tsong-Hai Lee, Shinichiro Uchiyama, Yohanna Kusuma, Hou Chang Chiu, Jose C. Navarro, Kay Sin Tan, Jeyaraj Pandian, Liang Guo, Yoko Wong, Narayanaswamy Venketasubramanian, for the Asian Stroke Advisory Panel

**Affiliations:** ^1^Linkou Chang Gung Memorial Hospital, Taoyuan, Taiwan; ^2^Clinical Research Center for Medicine, International University of Health and Welfare, Center for Brain and Cerebral Vessels, Sanno Medical Center, Tokyo, Japan; ^3^National Brain Center Hospital, Jakarta, Indonesia; ^4^Taipei Medical University-Shuang Ho Hospital, Taipei, Taiwan; ^5^Jose R. Reyes Memorial Medical Center, Manila, Philippines; ^6^University of Malaya Medical Center, Kuala Lumpur, Malaysia; ^7^Christian Medical College and Hospital, Ludhiana, India; ^8^Singapore Clinical Research Institute, Consortium for Clinical Research and Innovation, Singapore, Singapore; ^9^Raffles Hospital, Singapore, Singapore

**Keywords:** stroke, recovery, neuro-restoration, review, rehabilitation, evidence

## Abstract

**Background:**

Stroke burden is largely due to long-term impairments requiring prolonged care with loss of productivity. We aimed to identify and assess studies of different registered pharmacological therapies as treatments to improve post-stroke impairments and/or disabilities.

**Methods:**

We performed a systematic-search-and-review of treatments that have been investigated as recovery-enhancing or recovery-promoting therapies in adult patients with stroke. The treatment must have received registration or market authorization in any country regardless of primary indication. Outcomes included in the review were neurological impairments and functional/disability assessments. “The best available studies” based on study design, study size, and/or date of publication were selected and graded for level of evidence (LOE) by consensus.

**Results:**

Our systematic search yielded 7,801 citations, and we reviewed 665 full-text papers. Fifty-eight publications were selected as “the best studies” across 25 pharmacological classes: 31 on ischemic stroke, 21 on ischemic or hemorrhagic stroke, 4 on intracerebral hemorrhage, and 2 on subarachnoid hemorrhage (SAH). Twenty-six were systematic reviews/meta-analyses, 29 were randomized clinical trials (RCTs), and three were cohort studies. Only nimodipine for SAH had LOE A of benefit (systematic review and network meta-analysis). Many studies, some of which showed treatment effects, were assessed as LOE C-LD, mainly due to small sample sizes or poor quality. Seven interventions had LOE B-R (systematic review/meta-analysis or RCT) of treatment effects.

**Conclusion:**

Only one commercially available treatment has LOE A for routine use in stroke. Further studies of putative neuroprotective drugs as adjunctive treatment to revascularization procedures and more confirmatory trials on recovery-promoting therapies will enhance the certainty of their benefit. The decision on their use must be guided by the clinical profile, neurological impairments, and target outcomes based on the available evidence.

**Systematic review registration:**

https://www.crd.york.ac.uk/prospero/display_record.php?RecordID=376973, PROSPERO, CRD42022376973.

## Introduction

Stroke is a major cause of death and disability with only a limited number of treatment options to improve functional outcomes or reduce death and disability after a stroke, including thrombolytic therapy, thrombectomy, early use of anti-platelets, decompression craniectomy for “malignant” infarcts, organized stroke care, and constraint-induced movement therapy (1). However, many patients do not receive time-sensitive acute stroke therapies for various reasons (2, 3). Alternative strategies using neuroprotectants have failed to live up to their earlier promise (4). Drug interventions that mediate recovery beyond the acute windows are, therefore, clinically important research targets.

As much as three-quarters of all stroke patients suffer impairments and disabilities, the most common of which are motor weakness (77.4%), urinary incontinence (48.2%), impaired consciousness (44.7%), dysphagia (44.7%), and impaired cognition (43.9%) (5). Transition from independence in activities of daily living to dependency between 3 and 12 months after a stroke may be observed in a high proportion of patients (6). At 5 years, functional and motor outcomes may deteriorate to the status at 2 months post-stroke (7). In a large multi-center clinical trial of stroke patients with one-third of participants coming from Asia, at a median follow-up of 4 years, 19–22% were disabled and 12–14% were dependent, requiring regular help with everyday activities (8).

Stroke burden is largely due to long-term impairments suffered after a stroke, requiring long-term care and loss of productivity (9–14). Improving the degree and chances of recovery will translate to an overall reduction in the burden and cost of stroke care. Apart from standard rehabilitation strategies, however, there is currently no common recommendation on pharmacological treatment for stroke recovery.

With the aging of the global population, the number of disabled stroke survivors is likely to rise. Clearly, treatments are needed to enhance recovery after stroke. Prematurely judging a treatment as ineffective may mean lost opportunities in moving stroke recovery research forward to benefit stroke sufferers. It is entirely possible that the apparent “lack” of the efficacy of neuro-recovery interventions thus far may not only be due to small sample sizes or varying severity of study subjects but also because of premature summative assessments and that following up at an extended time frame might show positive effects. Conversely, claiming a treatment as effective, when there is a lack of evidence, can be problematic as patients may be exposed unnecessarily to possible side effects or miss the opportunity of receiving a more appropriate treatment, in addition to incurring the costs of an ineffective intervention. A review of registered pharmacological therapies that have been investigated for improving post-stroke outcomes will help identify the types of available evidence, information on how research was conducted on them, key characteristics or factors related to treatment effects, and knowledge gaps in the pharmacological treatment of post-stroke patients that will be helpful in both clinical decision-making and planning future studies.

We, therefore, aimed to identify and assess studies of different registered pharmacological therapies investigated for improving post-stroke impairments and/or disabilities. The research questions we sought to answer are:

1. What is the best available evidence based on study design for different registered pharmacological therapies investigated for improving recovery after a stroke?
2. What stroke sub-populations and post-stroke outcomes are improved by these treatments, if any.

## Methods

This systematic-search-and-review (15) was registered in the International Prospective Register of Systematic Reviews in 2022 (PROSPERO CRD42022376973).

### Eligibility criteria

Studies that have the following PICO study characteristics were included in the review:

#### Participants (P)

Studies examining adult humans, aged 18 years or older, diagnosed with stroke were included. Studies addressing both adults and children were included if data provided for adults were reported separately.

#### Interventions (I)

Interventions were pharmacological therapies that have been investigated as recovery-enhancing or recovery-promoting treatments in patients who had suffered a stroke. These treatments must have received registration or market authorization in any country, either prescription or over-the-counter products, and may have primary indications for use in other medical conditions. Supplementary Table S1 lists the pharmacological classes and drugs investigated in the review.

#### Comparators (C)

Depending on the study type, a comparator (active or placebo) may have been included.

#### Outcomes (O)

Stroke-related outcomes included overall function and motor recovery. Other clinical neurological domains were also considered. The following outcomes were excluded:

- Psychiatric—mood (e.g., depression, mania, anxiety, and apathy), sleep disorder, hypersexuality, emotionalism, delirium, etc.
- Cognitive—dementia, memory impairment, concentration, neglect, etc.
- Spasticity, contracture, sialorrhea, seizures, pain, and fatigue.
- Imaging and laboratory outcomes (e.g., lesion size, vasospasm, biomarkers, and transcranial magnetic stimulation parameters).

### Search strategy

A literature search was carried out in PubMed, EMBASE, Scopus, the Cochrane Database of Systematic Reviews, the Cochrane Central Register of Controlled Trials (CENTRAL), and the Database of Abstracts of Reviews of Effects for published reports up to November 2022. The search was also supplemented by searching for trial protocols at https://www.clinicaltrials.gov and completing systematic reviews in PROSPERO. The search criteria included (i) both MeSH terms and free text related to “stroke” and “recovery”; (ii) each of the pharmacological classes/products listed, (iii) limited to the English language, and (iv) human subjects only. The search strategy for EMBASE is shown in Supplementary Table S2. The search syntax was adjusted accordingly in each search engine with the same criteria. To ensure literature saturation, the reference lists of included studies or relevant reviews were scanned.

### Selection of sources of evidence

Literature search results were uploaded to Covidence^1^ to facilitate collaboration among reviewers during the study selection process. The search results were grouped by pharmacological class. Duplicates were identified and removed automatically by Covidence and by manual checking. Titles and abstracts screening was conducted by at least one author, and only relevant studies were further retrieved and reviewed in the full text of the publication. Included studies were classified into one of the categories in decreasing level of evidence: systematic review and/or meta-analysis, randomized controlled trial (RCT), non-randomized controlled trial, cohort study, case–control study, case report, or opinion of expert(s).

The decision on selecting “the best available studies” based on study design was made by two reviewers. If multiple papers were identified under the same hierarchy, a decision was reached by consensus based on study size and date of publication for different stroke subtypes and/or outcomes. Any disagreement on study selection and data extraction was resolved by consulting a third author for arbitration.

### Data extraction

Data extraction was performed using a pre-designed form for each included report. In cases of ambiguity of information, the study was elevated for adjudication by arbitrators.

Data extracted included patient demographics (age, gender, and country of origin), methodology (study design, sample size, and key stroke inclusion criteria), intervention details (dosage, frequency, duration of intervention, and type of control used), duration (stroke onset to study inclusion and follow-up period), and the reported outcomes (as dichotomous or continuous). If the outcome was reported as a composite measure, individual outcomes reported in the studies were extracted, if available. Whenever possible, we used results from an intention-to-treat analysis. For cross-over trials, we extracted data from the first period only to avoid any possible carry-over effects or the potential for stroke patients to recover spontaneously during cross-over.

### Synthesis of results

The included studies were grouped by pharmacological class/product and stroke subtypes in the results, with short narratives highlighting the important points. The level of evidence was assessed according to the latest version of the American Stroke Association Level of Evidence (LOE) scheme (Table 1) (16). Individual studies were given only one level of evidence, but systematic reviews and meta-analyses may be given several levels of evidence for different analyses performed.

**Table 1:** American Stroke Association level of evidence scheme (16).

Level of evidence	Definition
A	High-quality evidence from more than 1 RCT. Meta-analyses of high-quality RCTs. One or more RCTs corroborated by high-quality registry evidence.
B-R (randomized)	Moderate quality evidence from 1 or more RCT. Meta-analyses of moderate-quality RCTs.
B-NR (nonrandomized)	Moderate quality evidence from 1 or more well-designed, well-executed nonrandomized studies, observational studies, or registry studies. Meta-analyses of such studies.
C-LD (limited data)	Randomized or nonrandomized observational or registry studies with limitations of design or execution. Meta-analyses of such studies. Physiological or mechanistic studies in human subjects.
C-EO (expert opinion)	Consensus of expert opinion based on clinical experience.

## Results

**Figure 1:**
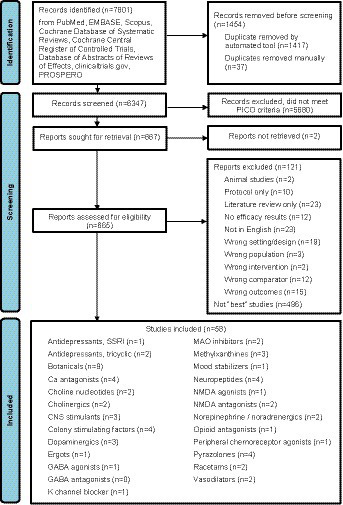
PRISMA flow diagram of review.

The systematic search yielded a total of 7,801 citations, of which 1,454 were duplicates. Of the remaining 6,347 papers screened by title and abstract, 5,680 did not meet one or more of the PICO criteria, while two papers could not be retrieved. After reviewing 665 full-text papers, a total of 58 publications were selected for inclusion as “the best” current studies across the different pharmacological classes (Figure 1).

Of the 58 publications included, 31 studies included patients with ischemic stroke, 21 on either “stroke” or “ischemic or hemorrhagic stroke,” four on intracerebral hemorrhage (ICH), and two on subarachnoid hemorrhage (SAH). Twenty-six were systematic reviews and/or meta-analyses, 29 were RCTs, and three were cohort studies. Among the included RCTs, the treatment window from stroke onset was up to 2 h (in one study), 5 h (one), 24 h (two), 48 h (four), 72 h (one), 96 h (one), 1 week (one), 2 weeks (one), 3 weeks (one), 1 month (three), 42 days (one), 3 months (one), 6 months (three), and > 6 months or “convalescing” or “chronic” (eight).

The characteristics of the 58 included studies are listed in Supplementary Table S3. The efficacy results of each study with their corresponding LOE (16) are summarized in Table 2, while the safety results are summarized in Table 3.

**Table 2:** Summary of efficacy results of included studies and assessed level of evidence according to the American Stroke Association scheme.

Treatment	Stroke subtype	Study	Study design	Study *N*	Outcomes	Time-points	Reached statistical threshold for benefit	Did not reach statistical threshold for benefit	LOE
**Antidepressants (selective serotonin reuptake inhibitors)**									
Fluoxetine, sertraline, paroxetine, citalopram, and escitalopram	Ischemic, ICH	Legg, 2021	SR/MA	13,029 (76 RCTs)	Impairment (NIHSS, etc.)	At the end of treatment		Low ROB studies: SMD −0.39, 95% CI −1.12–0.33; *p* = 0.29 (one study, fluoxetine, *n* = 30)	C-LD
					Motor deficit (FMA, SIS strength, etc.)	At the end of treatment		Low ROB studies: SMD 0.03, 95% CI −0.02 to 0.08; *p* = 0.23 (six studies, fluoxetine, *n* = 5,518, *I*^2^ = 75%)	A
					Independence (“typically mRS 0 to 2”)	At the end of treatment		Low ROB studies: RR 0.98, 95% CI 0.93–1.03; *p* = 0.37 (five studies, all fluoxetine, *n* = 5,926, *I*^2^ = 32%)	A
							All studies: citalopram RR 0.90, 95% CI 0.82–0.98; *p* = 0.01 (one study, *n* = 642)	All studies: RR 0.97, 95% CI 0.93–1.01; *p* = 0.18 (eight studies, *n* = 6,792, *I*^2^ = 59%)	B-R
								Fluoxetine RR 0.98, 95% CI 0.94–1.03; *p* = 0.50 (six studies, *n* = 6,039, *I*^2^ = 56%)	
								Sertraline RR 1.00, 95% CI 0.9–1.04; *p* = 1.00 (one study, *n* = 111)	
					Disability (BI, FIM, SIS, ADL, etc.)	At the end of treatment		Low ROB studies: SMD 0.00, 95% CI −0.05 to 0.05; *p* = 0.98 (five studies, all fluoxetine, *n* = 5,436, *I*^2^ = 0%)	A
							All studies: SMD −0.18, 95% CI −0.23 to −0.14; *p* < 0.00001 (32 studies, *n* = 7,667, *I*^2^ = 94%)		B-R
							Fluoxetine SMD −0.09, 95% CI −0.13 to −0.04; *p* = 0.006 (19 studies, *n* = 6,590, *I*^2^ = 87%)		
							Sertraline SMD −1.38, 95% CI −1.76 to −0.99; *p* < 0.00001 (one study, *n* = 130)		
							Paroxetine SMD −1.29, 95% CI -1.55 to −1.03; *p* < 0.00001 (five studies, *n* = 293, *I*^2^ = 85%)		
							Citalopram SMD −0.68, 95% CI −0.88 to −0.48; *p* < 0.00001 (five studies, *n* = 446, *I*^2^ = 95%)		
							Escitalopram SMD −0.67, 95% CI −1.00 to −0.34; *p* < 0.0001 (two studies, *n* = 208, *I*^2^ = 99%)		
**Antidepressants (tri- or tetracyclic)**									
Maprotiline	Ischemic	Dam, 1996	RCT	46	HSS	3 months		48.3 ± 13.8 vs. 46.8 ± 9.9 (n.s.)	C-LD
					HSS motor	3 months		32.2 ± 4.7 vs. 31.6 ± 5.0 (n.s.)
					HSS gait	3 months		4.8 ± 1.5 vs. 4.6 ± 1.3 (n.s.)
					BI	3 months		47.9 ± 15.5 vs. 54.1 ± 21.1 (n.s.)
					good recovery (HSS gait + BI)	3 months		5/14 (36%) vs. 6/16 (38%) (n.s.)
Nortriptyline	Ischemic, ICH	Mikami, 2011	RCT	83 (Ischemic 74, ICH 9)	mRS	3, 6, 9, and 12 months	Mixed model time-treatment interaction [*t* (105) = 2.91, *p* = 0.004] nortriptyline compared to placebo		C-LD
					FIM	3, 6, 9, 12 months	Mixed model time-treatment interaction [*t* (153) = 1.71, *p* = 0.0089] nortriptyline or fluoxetine compared to placebo	
**Botanicals**									
Di huang yin zi	Ischemic	Yu, 2015	RCT	87	FMA	4 weeks		MD 1.2, 95% CI −4.0–6.4	C-LD
						8 weeks	MD 7.0, 95% CI 1.6–12.4	
						12 weeks	MD 6.5, 95% CI 0.7–12.3	
					BI	4 weeks		MD 3.7, 95% CI −1.0–8.4
						8 weeks		MD 2.3, 95% CI −1.9–6.5
						12 weeks	MD 4.5, 95% CI 0.3–8.7	
Ginkgo biloba	Ischemic	Chong, 2020	SR/MA	1,466 (13 RCTs)	NIHSS	At the end of study	MD −2.87, 95% CI −4.01 to −1.74; *p* < 0.00001 (five studies, *n* = 467, *I*^2^ = 85%)		C-LD
					BI	At the end of study	MD 9.52, 95% CI 4.66–14.38; *p* = 0.00001 (three studies, *n* = 535, *I*^2^ = 87%)	
					mRS	At the end of study	MD −0.50, 95% CI −0.63 to −0.37; *p* < 0.00001 (two studies, *n* = 193, *I*^2^ = 0%)	
Ginkgo biloba	Ischemic	Ji, 2020	SR/MA	1,829 (15 RCTs)	NIHSS	“Acute”	MD −1.39, 95% CI −2.15 to −0.62; *p* = 0.0004 (three studies, *n* = 504, *I*^2^ = 0%)		C-LD
						“Convalescence”	MD: −1.15, 95% CI −1.76 to −0.53; *p* = 0.0003 (two studies, *n* = 424, *I*^2^ = 0%)	
					NFDS	“Acute”	Improved >18%: 279/303 vs. 233/304, RR 1.20, 95% CI 1.12–1.29; *p* < 0.00001 (seven studies, *n* = 607, *I*^2^ = 0%)	
						“Convalescence”	Improved >18%: 279/303 vs. 233/304, RR: 1.17, 95% CI 1.09–1.27; *p* < 0.0001 (four studies, *n* = 613, *I*^2^ = 0%)	
					BI	“acute”	MD 5.72, 95% CI 3.11–8.33; *p* < 0.0001 (two studies, *n* = 425, *I*^2^ = 29%)	
						“Convalescence”	MD 7.17, 95% CI 5.96–8.38, *I*^2^ = 0; *p* < 0.00001 (two studies, *n* = 407, *I*^2^ = 0%)	
MLC601/MLC901	Ischemic	Harandi, 2011	RCT	150	FMA	4 weeks	77.13 ± 19.22 vs. 63.50 ± 24.21; *p* < 0.001		B-R
						8 weeks	82.51 ± 14.27 vs. 72.06 ± 21.41; *p* = 0.001	
						12 weeks	86.22 ± 12.34 vs. 74.36 ± 18.1; *p* < 0.001	
MLC601/MLC901	Ischemic	González-Fraile, 2016	SR/MA	1,936 (5 RCTs)	BI ≥ 65, mRS 0–1, or DTER item #8 = 0	1 month	RR 2.40, 95% CI 1.28–4.51 (two studies, *n* = 605, *I*^2^ = 7.6%)		B-R
						3 months		RR 1.37, 95% CI 0.85–2.21 (three studies, *n* = 1,331, *I*^2^ = 83.6%)
						At the end of treatment (1 or 3 months)	RR 1.64, 95% CI 1.05–2.57; *p* = 0.031 (five studies, *n* = 1936, *I*^2^ = 80.2%)	
MLC601/MLC901	Ischemic	Venketasubramanian, 2015	RCT	880	mRS	6 months	mRS 0–1 OR 1.49, 95% CI 1.11–2.01		B-R
						12 months	mRS 0–1 OR 1.41, 95% CI 1.05–1.90
						18 months	mRS 0–1 OR 1.36, 95% CI 1.01–1.83
						24 months		Ordinal OR 1.08, 95% CI 0.85–1.37
								mRS 0–1 OR 1.29, 95% CI 0.96–1.74
					BI	6 months	BI ≥ 95 OR 1.55, 95% CI 1.14–2.10	
						12 months		BI ≥ 95 OR 1.22, 95% CI 0.89–1.66
						18 months		BI ≥ 95 OR 1.31, 95% CI 0.95–1.79
						24 months		BI ≥ 95 OR 1.36, 95% CI 0.99–1.86
MLC601/MLC901	ICH	Kumar, 2020	Cohort study	66	NIHSS	3 months	Mean baseline 12.7 ± 8.3, mean at 3 months 5.2 ± 6.6 (month 3 vs. baseline *p* < 0.0001)		C-LD
					GCS	3 months	Mean baseline 12.8 ± 3.1, mean at 3 months 14.5 ± 1.3 (month 3 vs. baseline *p* = 0.001)	
					mRS	3 months	mRS 0–1 baseline 6 (9.1%), mean at 3 months 15 (36.6%) (month 3 vs. baseline *p* = 0.004)	
Panax notoginseng	Ischemic	Chen, 2008	SR/MA	660 (8 RCTs)	Neurological deficit (NIHSS, CNS, ESS, SSS, mESSS, etc.)	At the end of treatment (14–28 days)	No improvement RR 0.29, 95% CI 0.18–0.47, *p* = 0.00001 (seven studies, *n* = 597, *I*^2^ = 0%)		C-LD
							mESSS MD -5.39, 95% CI −7.76 to −3.02; *p* < 0.00001 (two studies, *n* = 206, *I*^2^ = 0%)		
					Death or dependency (BI < 60, mRS 3–5, etc.)	At the end of follow-up (28 days)	RR 0.63, 95% Cl 0.45–0.88; *p* = 0.0072 (two studies, *n* = 165, *I*^2^ = 0.0%)	
Panax notoginseng	ICH	Xu, 2015	SR/MA	1,891 (20 RCTs)	“Effectiveness rate”	At the end of treatment (2, 3, or 4 weeks)	OR 2.70; 95% CI 2.16–3.38; *p* < 0.00001 (13 studies, *n* = 1,435, *I*^2^ = 0%)		B-R
					Neurological deficit score	At the end of treatment (7, 15, 21, 28, or 30 days)	MD 4.36; 95% CI 3.07–5.65; *p* < 0.00001 (six studies, *n* = 434, *I*^2^ = 0%)
					BI	At the end of treatment (14 or 28 days)	MD 11.73; 95% CI 19.31–4.16; *p* = 0.002 (two studies, *n* = 78, *I*^2^ = 61%)
**Calcium antagonists**									
Nimodipine, flunarizine, isradipine, nicardipine, and fasudil, lifarizine	Ischemic, ICH	Zhang, 2019	SR/MA	7,731 (34 RCTs; two studies included 225 ICH)	Death or dependency (mRS > 3, GOS < 4, BI < 60, TSS > 3, Mathew Impairment Scale = 7)	At the end of follow-up		Overall RR 1.05, 95% CI 0.98 to 1.13; *p* = 0.16 (22 studies, *n* = 6,684, *I*^2^ = 28.8%)	A
								Nimodipine RR 1.06, 95% CI 0.97 to 1.14; *p* = 0.19 (19 studies, *n* = 6,093,*I*^2^ = 32.26%)	
								Flunarizine RR 0.81, 95% CI 0.34 to 1.94; *p* = 0.19 (2 studies, *n* = 357, *I*^2^ = 64.44%)	
								Isradipine RR 1.01, 95% CI 0.74 to 1.4; *p* = 0.93 (1 study, *n* = 234)	
								Others RR 0.98, 95% CI 0.58 to 1.66 (3 studies, *n* = 370)	
Nimodipine, nicardipine, magnesium	SAH	Dayyani, 2022	SR/MA	5,234 (25 RCTs)	GOS	At the end of follow-up	GOS ≥ 4: nimodipine OR 1.46, 95% CI 1.07 to 1.99; absolute risk increase (ARI) 8.25, 95% CI 1.55 to 14.09	GOS ≥ 4: nicardipine OR 0.95, 95% CI 0.69 to 1.32	A
								Magnesium OR 1.29, 95% CI 0.95 to 1.75	
					mRS	At the end of follow-up	mRS ≤ 2: nicardipine OR 8.80, 95% CI 1.34 to 57.77; ARI 29.84, 95% CI 6.41 to 34.84	mRS ≤ 2: magnesium OR 1.01, 95% CI 0.77 to 1.34
Magnesium	Ischemic or hemorrhagic	Avgerinos, 2019	SR/MA	4,347 (7 RCTs)	mRS	90 days		mRS < 1: OR 1.05, 95% CI 0.92 to 1.20; *p* = 0.46 (3 studies, *n* = 4,111, *I*^2^ = 0%)	A
								SMD −0.25, 95% CI −0.50 to 0.00; *p* = 0.05 (6 studies, *I*^2^ = 82%)	
							Ischemic stroke only studies: WMD -0.96, 95% CI −1.34 to −0.58; *p* < 0.00001 (3 studies, *n* = 164, *I*^2^ = 0%)		C-LD
					BI	90 days		BI > 60: OR 1.05, 95% CI 0.92 to 1.19; *p* = 0.48 (4 studies, *n* = 4,171, *I*^2^ = 0%)	A
								BI > 95: OR 0.95, 95% CI 0.76 to 1.20; *p* = 0.70 (3 studies, *n* = 4,111, *I*^2^ = 54%)	
								SMD 0.03, 95% CI -0.07 to 0.13; P = 0.50 (6 studies, *I*^2^ = 20%)	
Magnesium	ICH	Naidech, 2022	RCT sub-analysis	268	NIHSS	90 days	6 (2 to 24) vs. 5 (2 to 21); *p* = 0.56	B-R
					mRS	90 days	mRS ≤ 2: 38 (26%) vs. 43 (35%); *P* = 0.16	
**Choline nucleotides**									
Citicoline	Ischemic	Martí-Carvajal, 2020	SR/MA	4,543 (10 RCTs)	NIHSS	6 weeks		NIHSS ≤ 1: RR 1.08, 95% CI 0.96 to 1.21; *p* = 0.19 (4 studies, *n* = 3,950, *I*^2^ = 27%)	A
					mRS	90 days	mRS < 3: RR 1.11, 95% CI 0.97 to 1.26; *p* = 0.13 (4 studies, *n* = 3,668, *I*^2^ = 1%)	A
					BI	90 days	BI > 50: RR 1.26, 95% CI 0.71 to 2.26; *p* = 0.43 (1 study, *n* = 77) BI ≥ 95: RR 1.03, 95% CI 0.94 to 1.13; *p* = 0.53 (4 studies, *n* = 2,850, *I*^2^ = 24%)	A
							BI ≥ 85: RR 3.13, 95% CI 1.10 to 8.91; *p* = 0.03 (1 study, *n* = 63)		C-LD
Citicoline + i.v. tPA or/and EVT	Ischemic	Agarwal, 2022	RCT	99	NIHSS	90 days		NIHSS ≤ 2: OR 0.96, 95% CI 0.39 to 2.40; *p* = 0.934	C-LD
					mRS	90 days	mRS ≤ 2: OR 0.92, 95% CI 0.40 to 2.05; *p* = 0.732	
					BI	90 days	BI ≥ 95: OR 0.87, 95% CI 0.22 to 2.98; *p* = 0.564	
**Cholinergics**									
Donepezil	“Stroke”	Berthier, 2006	RCT	26	Aphasia quotient of WAB	16 weeks	Mean change 6.4 ± 3.8, 95% CI 4.13 to 8.81 vs. 3.5 ± 2.7, 95% CI 1.93 to 5.22; *p* = 0.037; Cohen’s d = 0.87		C-LD
					Communicative Activity Log	16 weeks		Mean change 8.2 ± 9.3 vs. 2.4 ± 9.0, *p* = n.s.
					Picture naming on PALPA	16 weeks	Mean change 4.6 ± 5.8, 95% CI 0.1 to 5.0 vs. −1.0 ± 6.3, 95% CI −0.3 to 5.0; *p* = 0.025; Cohen’s d = 0.92	
					Auditory phonemic discrimination-word pairs	16 weeks		Mean change 3.4 ± 6.6 vs. 3.0 ± 7.3 (n.s.)
					Auditory lexical decision	16 weeks	Mean change 8.0 ± 15.5 vs. 1.5 ± 4.5; (n.s.)
					Word repetition	16 weeks	Mean change 0.7 ± 1.8 vs. 1.0 ± 2.9; (n.s.)
					Nonword repetition	16 weeks	Mean change −0.2 ± 5.2 vs. 2.4 ± 2.3; (n.s.)
					Picture naming	16 weeks	Mean change 4.6 ± 5.8 vs. −1.0 ± 6.3; (n.s.)
					Spoken word-picture matching	16 weeks	Mean change 2.6 ± 4.4 vs. 0.1 ± 3.9; (n.s.)
					Spoken sentence-picture matching	16 weeks	Mean change 2.6 ± 3.4 vs. 1.6 ± 3.5; (n.s.)
Pre-stroke donepezil, rivastigmine, or galantamine	Ischemic	Wakisaka, 2021	Cohort study	805	NIHSS	During hospitalization	≥2 points increase: multivariable OR 0.52, 95% CI 0.31 to 0.88; *p* = 0.01		B-NR
							Propensity matched: OR 0.47, 95% CI 0.25 to 0.86; *p* = 0.02		
					mRS	3 months	mRS ≥ 3: multivariable OR 0.68, 95% CI 0.46 to 0.99; *p* = 0.048	
							Propensity matched: OR 0.61, 95% CI 0.40 to 0.92; *p* = 0.02	
**Central nervous system stimulants**									
Amantadine, modafinil	Ischemic, ICH, SAH	Gagnon, 2020	SR	Amantadine *n* = 128 (10 studies)	Amantadine: 11 unique measures with 46 domains reported		Quantitative analyses not performed due to heterogeneity in outcome measures.		C-LD
							1 RCT (moderate quality) reported consistent improvements in the Coma Recovery Scale—revised and Disability Rating Scale on day 5, 3 months, and 6 months (all *p* < 0.03). None of the late post-stroke studies were of high quality; among the 4 control groups, only 2 showed improvement: increased word finding in 3 in an on–off–on–off study, and improved activity and intellectual, motor, and emotional function. 4 of 5 case reports improvement.		
				Modafinil *n* = 138 (12 studies)	Modafinil: 31 unique measures with 116 domains reported		Quantitative analyses not performed due to heterogeneity in outcome measures.		C-LD
							1 RCT (high quality) showed fatigue was not improved at 90 days (*p* = 0.3), although the FSS (*p* = 0.019), FSS-7 (*p* = 0.04), SS-QOL work/productivity (*p* = 0.007), language (P0.012), UE function (*p* = 0.02). 1 RCT (cross-over design) 3 to 38 months after stroke showed reduced fatigue (MFI-20, *p* ≤ 0.001), SS-QOL (*p* = 0.01) with 6 weeks treatment. In 2 later post-stroke studies with control groups, fatigue was reduced during 3 months of modafinil for diencephalon or brainstem strokes but not for cortical strokes. In 18 patients, 5 who continued modafinil for 1 year scored higher than 13 who did not. No difference in fatigue between groups in a retrospective study (very low quality). 6 of 7 case reports suggested improvement.		
Amphetamine	Ischemic	Goldstein, 2018	RCT	64	NIHSS	At the end of treatment		Mean 10.47 (SEM 0.98) vs. 9.86 (1.30); *p* = 0.476	B-R					
						3 months		Mean 8.61 (SEM 1.00) vs. 8.64 (1.01); *p* = 0.941					
								Mean change −4.84 (0.75) vs. -4.96 (0.77); *p* = 0.974					
					CNS	At the end of treatment		Mean (SEM) *p* = 0.645					
						3 months		Mean (SEM) *p* = 0.738					
								Mean change 1.95 (0.27) vs. 2.04 (0.33); *p* = 0.662					
					FMA	At the end of treatment		Mean 35.58 (SEM 4.41) vs. 38.5 (4.77); *p* = 0.63					
						3 months		Mean 42.10 (SEM 4.61) vs. 44.68 (4.89); *p* = 0.63					
								Mean change 18.65 (2.27) vs. 20.83 (2.94); *p* = 0.58					
					6-min Walk Test	At the end of treatment		Mean 367.59 (SEM 70.32) vs. 335.26 (73.56) ft.; *p* = 0.732						
						3 months		Mean 536.90 (SEM 95.21) vs. 422.12 (93.48) ft.; *p* = 0.478					
								Mean change 359.71 (70.07) vs. 222.13 (64.14) ft.; *p* = 0.156					
					ARAT	At the end of treatment		Mean 67.09 (SEM 4.40) vs. 71.46 (4.07); *p* = 0.271					
						3 months		Mean 70.62 (SEM 4.37) vs. 77.68 (4.58); *p* = 0.129					
								Mean change 7.24 (2.72) vs. 12.83 (3.69); *p* = 0.082					
					mRS	At the end of treatment		“not significant”					
						3 months		mean change 0.71 [SEM 0.14] vs. 0.92 (0.13); *p* = 0.29					
					FIM	At the end of treatment		Mean 85.63 (SEM 4.34) vs. 81.21 (4.17); *p* = 0.588					
						3 months		Mean 98.29 (SEM 5.04) vs. 97.12 (4.26); *p* = 0.597					
								Mean change 38.29 (3.31) vs. 34.46 (2.84); *p* = 0.461					
					SIS	3 months		Mean 53.93 (SEM 3.11) vs. 53.44 (2.74); *p* = 0.892					
								Mean change 18.04 (2.23) vs. 17.58 (3.49); *p* = 0.861
Methylphenidate	Ischemic	Lokk, 2011	RCT	78	NIHSS	3 months		MPH 2.9 ± 2.6 vs. LD 1.8 ± 2 vs. MPH + LD 3.7 ± 3 vs. placebo 4.0 ± 3.6 (*p* = 0.089)	C-LD					
						6 months	Mean change: MPH −3.3 ± 1.4 vs. LD −2.6 ± 1.2 vs. MPH + LD −3.6 ± 1.6 vs. placebo −1.9 ± 1.4 (*P* = 0.001)	MPH 2.6 ± 2.5 vs. LD 1.7 ± 1.9 vs. MPH + LD 3.5 ± 3 vs. placebo 3.6 ± 2.8 (*p* = 0.104)					
					FMA	3 months		MPH 57.0 ± 35.3 vs. LD 66.3 ± 31.7 vs. MPH + LD 57.7 ± 37.1 vs. placebo 53.4 ± 34.4 (*p* = 0.685)						
						6 months	MPH 58.0 ± 35.5 vs. LD 68.2 ± 31.4 vs. MPH + LD 56.9 ± 35.5 vs. placebo 54.4 ± 34.2 (P = 0.597)					
							Mean change: MPH 19.7 ± 13.7 vs. LD 21.8 ± 12.2 vs. MPH + LD 23.1 ± 19 vs. placebo 13.3 ± 12.7 (*p* = 0.169)					
					BI	3 months	MPH 71.58 ± 16 vs. LD 76.75 ± 12.4 vs. MPH + LD 72.37 ± 14.4 vs. placebo 70.50 ± 14.4 (*p* = 0.548)					
						6 months	Mean change: MPH 25.5 ± 14.2 vs. LD 30 ± 18.9 vs. MPH + LD 30.5 ± 13.3 vs. placebo 16.5 ± 9.6 (*p* = 0.011)	MPH 77.4 ± 14.5 vs. LD 84.5 ± 8.5 vs. MPH + LD 83.2 ± 15.4 vs. placebo 73.25 ± 14.1 (*p* = 0.343)
**Colony stimulating factors**									
EPO, GCSF	Ischemic, ICH	Bath, 2013	SR/MA	1,275 (11 RCTs)	NIHSS	At the end of treatment		EPO MD −2.20, 95% CI −10.01 to 5.61; *p* = 0.58 (1 study, *n* = 40).	C-LD	GCSF MD −0.40, 95% CI −1.82 to 1.01, *p* = 0.58 (5 studies, *n* = 203, *I*^2^ = 0%)	A
							Death or dependency (mRS or BI)	At the end of follow-up	EPO OR 1.01, 95% CI 0.72 to 1.42; *p* = 0.94 (2 studies, n = 562, *I*^2^ = 0%)	A
					GCSF OR 1.28, 95% CI 0.88 to 1.85; *p* = 0.20 (7 studies, *n* = 500, *I*^2^ = 0%)					
					EPO, GCSF	Ischemic, ICH	Chen, 2021	SR/MA	485 (8 RCTs)	NIHSS	At the end of follow-up	SMD −0.40, 95%CI −0.93 to 0.13 (6 studies, *I*^2^ = 79.7%)	A
BI	At the end of follow-up	SMD 0.04, 95%CI −0.38 to 0.46 (5 studies, *I*^2^ = 54.3%)	A						
GCSF	Ischemic, ICH	Huang, 2017	SR/MA	1,037 (14 RCTs)	NIHSS	3 months	MD −0.16, 95% CI −1.02 to 0.70; *p* = 0.72 (8 studies, *n* = 563, *I*^2^ = 92%)	A
					BI	3 months	MD 8.65, 95% CI 0.98 to 16.32; *p* = 0.03 (6 studies, *n* = 171, *I*^2^ = 79%)		C-LD
EPO + human choriogonadotropin	Ischemic	Cramer, 2014	RCT dose-escalation study	96	NIHSS	90 days		Median (IQR) change: low dose −8 (−9.75 to 3.25), medium dose −7·5 (−10 to −11.5), high dose −6 (−9 to −5); all active −8 (−9 to −3.25) vs. placebo −8 (−10 to −5); *p* = 0.31	C-LD
					mRS	90 days	“no significant difference between groups”
					BI	90 days	“no significant difference between groups”
**Dopaminergics**									
Levodopa	Ischemic, ICH	Ford, 2019	RCT	593	RMI ≥7 + able to walk ≥10 m	8 weeks		OR 0.78, 95% CI 0.53 to 1.15; *p* = 0.212	B-R					
					RMI	8 weeks	Adjusted MD −0.35, 95% CI −0.89 to 0.19; *p* = 0.198
						6 months	Adjusted MD 0.14, 95% CI −0.50 to 0.7; *p* = 0.662
						12 months	Adjusted MD 0.17, 95% CI −0.54 to 0.88; *p* = 0.637
					Nottingham Extended Activities Daily Living	8 weeks	Adjusted MD 1.02, 95% CI −1.27 to 3.30; *p* = 0.382
						6 months	Adjusted MD 0.027, 95% CI −2.72 to 2.78; *p* = 0.985
						12 months	Adjusted MD 1.04, 95% CI −1.56 to 3.64; *p* = 0.434
					BI	8 weeks	Adjusted MD −0.22, 95% CI −0.87 to 0.43; *p* = 0.511
						6 months	Adjusted MD −0.33, 95% CI −1.08 to 0.41; *p* = 0.378
						12 months	Adjusted MD −0.22. 95% CI −1.04 to 0.59; *p* = 0.591
					Manual ability measure	8 weeks	adjusted MD −0.10, 95% CI −0.46 to 0.26; *p* = 0.585
						6 months	Adjusted MD −0.15, 95% CI −0.57 to 0.27; *p* = 0.478
						12 months	Adjusted MD −0.16, 95% CI −0.59 to 0.28; *p* = 0.479					
					mRS	8 weeks		OR 0.87, 95% CI 0.63 to 1.21; *p* = 0.404					
						6 months	OR 0·81, 95% CI 0.57 to 1.14; *p* = 0.226
Ropinirole	Ischemic, ICH	Cramer, 2009	RCT	33	FMA	12 weeks	Time × group interaction term (repeated-measures ANOVA) was not significant	C-LD
					Gait velocity	12 weeks	Time × group interaction term (repeated-measures ANOVA) was not significant (data shown in graph)
					Gait endurance	12 weeks	Time × group interaction term (repeated-measures ANOVA) was not significant
					BI	12 weeks	Time × group interaction term (repeated-measures ANOVA) was not significant
					SIS-16	12 weeks	Time × group interaction term (repeated-measures ANOVA) was not significant
Bromocriptine, pergolide, pramipexole, carbidopa/levodopa, amantadine	Ischemic, ICH	Conroy, 2005	Cohort study	919	Rehabilitation length of stay	At discharge	Moderate stroke 18.0 vs. overall 15.2 (+2.8) days (*p* = 0.01 to 0.05) (worse)	B-NR
							Severe stroke 28.4 vs. overall 24.9 (+3.5) days (*p* = 0.001 to 0.01) (worse)	
					FIM	At discharge	Moderate stroke 18.1 vs. overall 22.4 (−4.3) (*p* < 0.001)
							Severe stroke 22.8 vs. overall 24.5 (−1.7) (*p* = 0.001 to 0.01)
**Ergots**									
Hydergine	“Stroke”	Bochner, 1973	RCT + cross-over	21 in RCT	“motor function”	12 weeks		MRC change arm +1.04 vs. +0.90 (*p* > 0.10)	C-LD
								MRC change leg +1.36 vs. +0.85 (*p* > 0.10)	
								Hand grips 10 s + 3.15 vs. +3.10 (*p* > 0.10)	
								Elbow flexion 10 s + 2.22 vs. +2.70 (*p* > 0.10)	
								Walk 12 ft. +5 vs. +6 (*p* > 0.10)	
								Time to sit up +4 vs. +6 (*p* > 0.10)	
								Time to drink 90 mL water −0.4 vs. −5.3 s (*p* > 0.10)							
						18 weeks		MRC change arm +0.16 vs. −0.05 (n.s.)	
								MRC change leg −0.01 vs. +0.15 (n.s.)	
								Dynamometer change −3.1 vs. +14.2 (n.s.)	
								Hand grips 10 s − 0.30 vs. +0.09 (n.s.)	
								Elbow flexion 10 s + 0.35 vs. +0.56 (n.s.)	
								Walk 12 ft. −3.17 vs. −0.53 (n.s.)	
								Time to sit up +0.17 vs. +1.43 (n.s.)	
**Gamma-aminobutyric acid (GABA) agonists**									
Clomethiazole, diazepam	Ischemic, ICH	Liu, 2018	SR/MA	3,838 (5 RCT)	NIHSS	3 months		CLASS-H (ICH, *n* = 198): mean change −4.5 vs. −4.0 (*p* = 0.36)	B-R
								CLASS-I (ischemic, *n* = 1,169): median (IQR) change −5.5 (−11, 17) vs. -6.0 (−10, 16) (*p* = 0.68)	
					SSS	3 months	No difference in total score (*p* = 0.56, 0.06, 0.23) or in motor power score (*p* = 0.96) (3 studies)	A
					BI > 60 or mRS < 3	3 months	Overall: RR = 1.0, 95% CI 0.94 to 1.06; *p* = 0.93 (5 studies, 3,758, *I*^2^ = 0%)	A
							Chlormethiazole RR 0.98, 95% CI 0.92 to 1.05; *p* = 0.57 (4 studies, *n* = 2,909, *I*^2^ = 0%)	
							Diazepam RR 1.07, 95% CI 0.93 to 1.22; *p* = 0.36 (1 study, *n* = 849)	
							Ischemic: RR 1.0, 95% CI 0.93 to 1.08; *p* = 0.92 (4 studies, *n* = 3,394, *I*^2^ = 18.31%)	
							ICH: RR 0.97, 95% CI 0.81 to 1.16; *p* = 0.74 (3 studies, *n* = 387, *I*^2^ = 0%)	
							Total Anterior Circulation Syndrome: RR 1.33, 95% CI 1.08 to 1.63; *p* = 0.01 (2 studies, *n* = 635, *I*^2^ = 0%)		B-R					
					Death or dependency (BI ≤ 60 or mRS ≥ 3)	3 months		Overall: RR 1.01, 95% CI 0.95 to 1.08; *p* = 0.78 (5 studies, *n* = 3,758, *I*^2^ = 0%)	A
								Clomethiazole: RR 1.03, 95% CI 0.96 to 1.11; *p* = 0.41 (4 studies, *n* = 2,909, *I*^2^ = 0%)	
								Diazepam: RR 0.94, 95% CI 0.82 to 1.07; *p* = 0.36 (1 study, *n* = 849)	
								ICH: RR 0.99, 95% CI 0.75 to 1.30; *p* = 0.92 (2 studies, n = 292, *I*^2^ = 0%)	
								Ischemic: RR 1.04, 95% CI 0.96 to 1.12; *p* = 0.35 (4 studies, *n* = 2,646, *I*^2^ = 0%)	
**Methylxanthines**									
Aminophylline	Ischemic	Bath, 2004a	SR/MA	119 (2 RCTs)	Death or neurological deterioration	4 weeks		OR 0.87, 95% CI 0.41 to 1.88, *p* = 0.73 (2 studies, *n* = 119, *I*^2^ = 0%)	B-R
					Death or disability	At the end of follow-up	OR 0.64, 95% CI 0.24 to 1.68; *p* = 0.36 (1 study, *n* = 73)	C-LD
Pentoxifylline, propentofylline	Ischemic	Bath, 2004b	SR/MA	793 (5 RCTs)	Death or disability	At the end of follow-up	OR 95% CI 0.49, 95% CI 0.20 to 1.20; *p* = 0.10 (2 studies, *n* = 200, *I*^2^ = 0%)	B-R
Theophylline + thrombolysis	Ischemic	Modrau, 2020	RCT	64	NIHSS	24 h	Mean change unadjusted MD -3.4, 95% CI −6.7 to −0.1, *p* = 0.044; adjusted MD -3.6, 95% CI −7.1 to −0.1; P = 0.043	>50% improvement: unadjusted OR 2.6, 95% CI 0.9 to 7.5, *p* = 0.070; adjusted OR 3.0, 95% CI 1.0 to 9.0; *p* = 0.056	C-LD
					mRS	90 days		mRS 0–1: unadjusted OR 1.1, 95% CI 0.4 to 3.1, *p* = 0.802; adjusted OR 1.3, 95% CI 0.4 to 4.0; *p* = 0.640.	
								Ordinal OR 1.44, 95% CI 0.58 to 3.59; *p* = 0.432	
**Monoamine oxidase (MAO) inhibitors**									
Moclobemide	Ischemic, ICH	Laska, 2005	RCT	89	ANELT and Reinvang’s “Grunntest for afasi” coefficient	6 months		Overall (*n* = 76) and Completers (*n* = 65): no differences between moclobemide and placebo groups (values only shown in graphs)	C-LD
						12 months	No further recovery from aphasia compared to 6 months (*n* = 56)	
Selegiline	Ischemic, ICH	Bartolo, 2015	RCT	47	FIM	2 weeks		Median 63 (IQR 43 to 72) vs. 56 (52 to 76); *p* = n.s.	C-LD
						6 weeks		Median 93 (IQR 74 to 112) vs. 76 (66 to 105); *p* = n.s.	
**Mood stabilizers**									
Lithium	Ischemic	Mohammadianinejad, 2014	RCT	66	Modified NIHSS	30 days	Mean change: cortical: −4.30 ± 1.88 (*n* = 13) vs. -2.00 ± 1.24 (*n* = 14); *p* = 0.003	Mean change: −2.34 ± 2.13 (*n* = 32) vs. -1.64 ± 1.25 (*n* = 34); *p* = 0.402	C-LD
								Noncortical: −1.00 ± 0.88 (n = 19) vs. −1.40 ± 1.23 (n = 20); *p* = 0.336	
					Hand subsection of FMA	30 days	Mean change: cortical: 4.00 ± 0.70 (*n* = 13) vs. 1.42 ± 1.91 (*n* = 14); *p* = 0.003	Mean change: 2.34 ± 2.00 (*n* = 32) vs. 1.50 ± 2.17 (*n* = 34); *p* = 0.070	
							>25% regained: 14 (43.8%) vs. Placebo 5 (14.7%); *p* = 0.009	Noncortical: 1.21 ± 1.81 (*n* = 19) vs. 1.55 ± 2.39 (n = 20); *p* = 0.647	
							Cortical: 11 (84.6%) vs. 3 (21.4%); *p* = 0.002	>25% regained: noncortical: 3 (15.8%) vs. 2 (10%); *p* = 0.661	
**Neuropeptides**									
cerebrolysin	Ischemic	Ziganshina, 2020	SR/MA	1,601 (7 RCTs)	death or dependency	at the end of follow-up		Data not available for analysis	A
cerebrolysin	Ischemic	Bornstein, 2018	SR/MA	1879 (9 RCTs)	NIHSS	21 or 30 days	MW 0.60, 95% CI 0.56 to 0.64; *p* < 0.0001 (9 studies, *n* = 1,879)		B-R
							NIHSS ≥ 4 OR 1.60, 95% CI 1.03 to 2.48; *p* = 0.035 (5 studies, *n* = 1705, *I*^2^ = 63.73%)		
					mRS	90 days	(Only NIHSS > 12 at baseline) MW 0.61, 95% CI 0.52 to 0.69; *p* = 0.01; MD 0.39, 95% CI 0.06 to 0.71; *p* = 0.02 (3 studies, *n* = 314, *I*^2^ = 0%)		
Cerebrolysin + rehabilitation	Ischemic	Chang, 2016	RCT	66	FMA	21 days, 2 months, 3 months	Subgroup with FMA < 50 at baseline (data shown only in graphs: ANOVA for FMA total F_3,102_ = 4.596, *p* < 0.05; FMA arm F_3,102_ = 3.605, *p* < 0.05)	Overall (data shown only in graphs): ANOVA no significant interaction effect between time and type of intervention for total, arm, or leg scores. No differences in improvement of scores at 3 months.	C-LD
Cerebrolysin	SAH	Woo, 2020	RCT	50	GOSE	3 months		GOSE 5 to 8: 15/25 vs. 16/25 (n.s.)	C-LD
						6 months	GOSE 5 to 8: OR 1.49, 95% CI 0.43 to 5.17; ordinal analysis *p* = 0.80	
					mRS	3 months	mRS 0 to 3: 18/25 v. 17/25 (n.s.)
						6 months	mRS 0 to 3: OR: 3.45; 95% CI 0.79 to 15.01; ordinal analysis *p* = 0.76
					BI	6 months	Mean: 91 ± 28 vs. 90 ± 24; (n.s.)
							BI ≥ 75: 23/25 vs. 18/25; (n.s.)
**N-Methyl-D-Aspartate (NMDA) agonists**									
Cycloserine	Ischemic, ICH	Cherry, 2014	RCT	20	Stability platform balance task	3 days		(means of each group before and after presented in graphs) ANOVA *F* = 0.00, df = 1, *p* = 0.993	C-LD
					Spooning beans	3 days	(means of each group before and after presented in graphs) ANOVA *F* = 0.429, df = 1, *p* = 0.521
					Associative recognition task	3 days	(means of each group before and after presented in graphs) ANOVA *F* = 0.159, df = 1, *p* = 0.695
					Single-leg stance on the balance beam	3 days	(means of each group before and after presented in graphs) ANOVA *F* = 0.067, df = 1, *p* = 0.798
**NMDA antagonists**									
Memantine	Ischemic	Beladi Moghadam, 2021	RCT	53	NIHSS	5 days	Mean change −2.96 ± 0.10 vs. −1.24 ± 0.96; *p* < 0.0001		C-LD
					BI	3 months	Mean change 6.00 ± 2.62 vs. 3.96 ± 1.76; *p* = 0.002	
Memantine + CIAT	Ischemic, ICH	Berthier, 2009	RCT	28	WAB	16 weeks	Aphasia Quotient 4.0 (SE 0.7) vs. 0.8 (0.5); *p* = 0.002	Spontaneous Speech 0.5 (0.2) vs. −0.7 (0.2); *p* = 0.077	C-LD
							Naming 0.7 (0.1) vs. 0.1 (0.1); *p* = 0 0.015	Auditory Comprehension 0.3 (0.1) vs. −0.1 (0.1); *p* = 0.086	
								Repetition 0.3 (0.2) vs. 0.5 (0.1); *p* = 0.988	
						18 weeks	Aphasia Quotient 8.5 (0.9) vs. 3.5 (0.8); *p* = 0.0001	Repetition 0.3 (0.1) vs. 0.6 (0.2); *p* = 0.764	
							Spontaneous Speech 2.2 (0.3) vs. 0.9 (0.4); *p* = 0.024		
							Auditory Comprehension 0.4 (0.1) vs. 0.0 (0.1); *p* = 0.037								
							Naming 1.2 (0.1) vs. 0.3 (0.2); *p* = 0.009			
						20 weeks	Aphasia Quotient 8.9 (1.1) vs. 4.3 (0.6); *p* = 0.005	Auditory Comprehension 0.4 (0.1) vs. 0.2 (0.9); *p* = 0.122
								Repetition 0.5 (0.2) vs. 0.8 (0.9); *p* = 0.969
							Spontaneous Speech 2.3 (0.4) vs. 1.2 (0.3); *p* = 0.035	Naming 1.0 (0.2) vs. 0.4 (0.1); *p* = 0.064
						24 weeks	Aphasia Quotient 6.0 (0.8) vs. 3.9 (0.8); *p* = 0.041	Spontaneous Speech 1.7 (0.4) vs. 1.1 (0.3); *p* = 0.119
								Auditory Comprehension 0.2 (0.1) vs. 0.1 (0.2); *p* = 0.354
								Repetition 0.3 (0.1) vs. 0.4 (0.2); *p* = 0.774
								Naming 0.7 (0.1) vs. 0.2 (0.2); *p* = 0.319
						48 weeks	Spontaneous Speech 2.7 (0.3) vs. 1.6 (0.2); *p* = 0.045	Aphasia Quotient 10.1 (1.0) vs. 7.2 (1.1); *p* = 0.083
								Auditory Comprehension 0.5 (0.1) vs. 0.1 (0.1); *p* = 0.233
								Repetition 0.4 (0.1) vs. 1.1 (0.2); *p* = 0.376
								Naming 1.3 (0.2) vs. 0.9 (0.2); *p* = 0.368
					Communicative Activity Log	16 weeks		3.2 (1.5) vs. 0.2 (1.4); *p* = 0.182
						18 weeks	6.0 (1.3) vs. 1.0 (2.2); *p* = 0.040	
						20 weeks	3.4 (2.0) vs. 0.7 (2.3); *p* = 0.142
						24 weeks	3.1 (1.8) vs. 0.7 (1.8); *p* = 0.269
						48 weeks	4.0 (2.0) vs. 1.0 (2.7); *p* = 0.289
**Norepinephrine/noradrenergics**									
Atomoxetine	Ischemic, ICH	Ward, 2017	RCT	12	FMA	At the end of treatment	MD of change 7.2, 95% CI 1.6 to 12.7; *p* = 0.016		C-LD
						1 month	MD of change 6.1, 95% CI −1.3 to 13.5; *p* = 0.10
					ARAT	At the end of treatment	MD of change 2.3, 95% CI −1.9 to 6.6; *p* = 0.25
						1 month	MD of change 3.6, 95% CI −2.8 to 10.0; *p* = 0.24
					Wolf Motor Function Test	At the end of treatment	MD of change 0.09, 95% CI −0.13–0.31; *p* = 0.39
						1 month	MD of change 0.10, 95% CI −0.14–0.34; *p* = 0.40
Reboxetine	“stroke”	Zittel, 2007	RCT cross-over study	10	Grip strength	1.5 h after treatment	ANOVA treatment vs. placebo *F* = 9.8, P = 0.003; affected vs. nonaffected hand: *F* = 6.65, *p* = 0.012; interaction *F* = 4.84, *p* = 0.031.		C-LD
					Hand tapping	1.5 h after treatment	ANOVA interaction drug × affected hand *F* = 4.04, *p* = 0.048	
					9-hole-peg test	1.5 h after treatment		“no difference”
**Opioid antagonists**									
Naloxone, nalmefene	Ischemic	Ortiz, 2021	SR	96 (4 studies)/ 916 (3 studies)	Neurological score, CNS, BI, NSS, GOS, NIHSS, GCS	Post-treatment, 10 days, 2 weeks, 20 days, 3 months	No statistical analysis performed because of different outcomes used.		C-LD
							1 of 4 studies on naloxone reported statistically significant results (*n* = 44, NSS baseline 61.50 ± 20 and 2 weeks 75.46 ± 16.23 vs. baseline 76.65 ± 11.13 and 2 weeks 82.10 ± 18.01; *p* < 0.01).		
							1 of 3 studies on nalmefene was statistically significant (*n* = 236, NIHSS at 20 days 17 ± 5 vs. 20 ± 5, *p* < 0.05; GCS at 10 days 9.5 ± 2.9 vs. 8.1 ± 2.7, *p* < 0.05)		
**Peripheral chemoreceptor agonists**									
Almitrine-raubasine	Ischemic	Li, 2004	RCT	74	NFDSBI	1 month	Mean 6.7 ± 4.7 vs. 9.6 ± 6.8, *p* = 0.038		C-LD
							Mean change 3.6 ± 3.2 vs. 1.9 ± 3.5, *p* = 0.034		
						2 months	Mean 4.9 ± 4.2 vs. 7.9 ± 5.7, *p* = 0.014	Mean change 5.4 ± 3.6 vs. 3.7 ± 4.2, *p* = 0.060 (0.013 Pearson)	
						3 months	Mean 4.2 ± 4.3 vs. 6.6 ± 5.6, *p* = 0.043	Mean change 6.1 ± 3.8 vs. 5.0 ± 4.7, *p* = 0.241 (0.023 Pearson)		1 month	Mean 88.6 ± 15.5 vs. 75.8 ± 29.3, *p* = 0.024		
					Mean change 14.6 ± 13.8 vs. 3.3 ± 14.2, *p* = 0.001								
					2 months	Mean 93.3 ± 14.4 vs. 81.3 ± 27.0, *p* = 0.021		
						Mean change 19.3 ± 13.6 vs. 8.8 ± 14.0, *p* = 0.002								
					3 months	Mean 96.6 ± 12.3 vs. 83.2 ± 28.0, *p* = 0.011							
						Mean change 22.6 ± 14.7 vs. 10.7 ± 17.0, *p* = 0.002		
					**Potassium channel blockers**									
Dalfampridine	Ischemic	Page, 2020	RCT	377	2-min Walk Test	12 weeks		>20% improvement: 17/121 (14.0%) vs. 23/121 (19%) vs. 17/126 (13.5%) (n.s.)	B-R
								Mean increase in distance (feet): 19.4 ± 39.6 vs. 20.4 ± 38.3 vs. 14.9 ± 40.0 (n.s.)	
					Walk-12	12 weeks		Mean 48.3 ± 25.4 vs. 49.3 ± 26.0 vs. 45.4 ± 23.0	
								Mean change −3.01 vs. -1.49 vs. -5.78 (n.s.)	
					Timed Up and Go	12 weeks		Mean change −0.48 ± 6.0 vs. -0.25 ± 7.4 vs. -0.40 ± 4.5 (n.s.)	
**Pyrazolones**									
Edaravone	Ischemic	Fidalgo, 2022	SR/MA	50,536 (19 studies)	mRS	3 months	mRS ≤ 1 (overall) OR 1.26, 95% CI 1.04 to 1.54; *p* = 0.02 (9 studies, *n* = 26,458, *I*^2^ = 53%)	mRS ≤ 1 (RCTs only) OR 1.43, 95% CI 0.92 to 2.22; *p* = 0.11 (3 studies, *n* = 336, *I*^2^ = 0%)	B-R
							mRS ≤ 2 (overall) OR 1.31, 95% CI 1.03 to 1.67; *p* = 0.03 (9 studies, *I*^2^ = 56%)	mRS ≤ 2 (RCTs only) OR 1.80, 95% CI 0.90 to 3.57; *p* = 0.09 (3 studies, *I*^2^ = 19%)	
Edaravone + i.v. tPA	Ischemic	Hu, 2021	SR/MA	1,877 (17 RCTs)	NIHSS	At the end of treatment	MD 3.95, 95% CI 2.92 to 4.99; *p* < 0.00001 (15 studies, *n* = 1719, *I*^2^ = 92%)		B-R
						7 days	MD 5.11, 95% CI 2.84 to 7.37; *p* < 0.00001 (6 studies, *n* = 741, *I*^2^ = 95%)	
						14 days	MD 3.11,95% CI 2.23 to 3.99; *p* < 0.00001 (9 studies, *n* = 1,182, *I*^2^ = 80%)	
Edaravone-dexborneol	Ischemic	Xu, 2021	RCT	1,194	NIHSS	14 days	Mean change MD −0.40, 95% CI -0.72 to −0.08; *p* = 0.01	NIHSS 0–1: OR 0.99, 95% CI 0.75 to 1.31, *p* = 0.93	B-R
						30 days		NIHSS 0–1: OR 1.12, 95% CI 0.88 to 1.41; *p* = 0.37
						90 days		NIHSS 0–1: OR 1.08, 95% CI 0.86 to 1.36; *p* = 0.51
					mRS	90 days	mRS ≤ 1: OR 1.42, 95% CI 1.12 to 1.81; *p* = 0.004	
							Common OR 1.28, 95% CI 1.04 to 1.57; *p* = 0.02	
					BI	14 days		BI ≥ 95: OR 1.02, 95% CI 0.80 to 1.28; *p* = 0.90
						30 days		BI ≥ 95: OR 1.06, 95% CI 0.84 to 1.33; *p* = 0.65
						90 days		BI ≥ 95: OR 1.13, 95% CI 0.88 to 1.44, *p* = 0.34
					SIS	90 days		MD 2.67, 95% CI -15.30 to 20.65; *p* = 0.77
Edaravone	ICH	Qin, 2022	SR/MA	3,454 (38 RCTs)	NIHSS	Not reported	MD −5.44 95% CI −6.44 to −4.44; *p* < 0.00001 (21 studies, *n* = 1,904, *I*^2^ = 95%)		C-LD
					BI	Not reported	MD 8.44 95% CI 7.65 to 9.23; *p* < 0.00001 (15 studies, *n* = 863, *I*^2^ = 6%)	
					“Total efficiency rate”	At the end of follow-up	RR 1.26 95% CI 1.22 to 1.31; *p* < 0.00001 (30 studies, *n* = 2,481, *I*^2^ = 1%)	
**Racetams**									
Piracetam	Ischemic	Ricci, 2012	SR/MA	1,002 (3 RCTs)	BI	12 weeks		BI < 85 OR 0.90, 95% CI 0.67 to 1.20; *p* = 0.47 (1 study, *n* = 723)	B-R
					Death or dependence (BI < 85)	12 weeks		OR 1.01, 95% CI 0.77 to 1.32; *p* = 0.95 (1 study, *n* = 923)	B-R
Piracetam	“stroke”	Zhang, 2016	SR/MA	261 (7 RCTs)	Global language performance	at the end of follow-up		SMD 0.23, 95% CI −0.03 to 0.49; *p* = 0.08 (7 studies, *I*^2^ = 47%)	C-LD
						<12 weeks	SMD 0.49, 95% CI 0.21 to 0.78; *p* = 0.0007 (5 studies, *n* = 199, *I*^2^ = 0%)	
						>12 weeks	SMD -0.15, 95% CI -0.54 to 0.24; *p* = 0.44 (3 studies, *n* = 103, *I*^2^ = 10%)
					Repetition	At the end of follow-up	SMD 0.13, 95% CI −0.17 to 0.44; *p* = 0.39 (5 studies, *I*^2^ = 12%)
						<12 weeks	SMD 0.19, 95% CI -0.13 to 0.50; *p* = 0.24 (4 studies, *n* = 159, *I*^2^ = 37%)
						>12 weeks	SMD 0.27, 95% CI -0.20 to 0.74; *p* = 0.26 (2 studies, *n* = 71, *I*^2^ = 0%)
					Naming ability	At the end of follow-up	SMD 0.23, 95% CI -0.08 to 0.54; *p* = 0.14 (5 studies, *I*^2^ = 0%)
						<12 weeks	SMD 0.23, 95% CI -0.09 to 0.54; *p* = 0.16 (4 studies, *n* = 159, *I*^2^ = 0%)
						>12 weeks	SMD 0.08, 95% CI -0.38 to 0.55; *p* = 0.72 (2 studies, *n* = 71, *I*^2^ = 0%)
					Written language	At the end of follow-up	SMD 0.35, 95% CI 0.04 to 0.66; *p* = 0.03 (5 studies, *I*^2^ = 0%)SMD 0.49, 95% CI 0.18 to 0.81; P = 0.002 (4 studies, *n* = 159, *I*^2^ = 0%)	
						<12 weeks	
						>12 weeks	SMD 0.20, 95% CI −0.27 to 0.67; *p* = 0.40 (2 studies, *n* = 71, *I*^2^ = 0%)
					Comprehension	At the end of follow-up	SMD 0.21, 95% CI -0.10 to 0.51; *p* = 0.19 (5 studies, *I*^2^ = 0%)
						<12 weeks	SMD 0.17, 95% CI -0.14 to 0.48; *p* = 0.29 (4 studies, *n* = 159, *I*^2^ = 0%)
						>12 weeks	SMD 0.30, 95% CI -0.17 to 0.77; *p* = 0.21 (2 studies, *n* = 71, **I*^2^* = 0%)
**Vasodilators**									
Buflomedil	Ischemic	Wu, 2015	SR/MA	2,756 (26 RCTs)	CSS, ESS	At the end of treatment	SMD −0.98, 95% CI −1.21 to −0.75; *p* < 0.0001 (seven studies, *n* = 745, *I*^2^ = 53%)		C-LD
					CSS	At the end of treatment	“Significant improvement”: RR 1.19, 95% CI 1.14–1.25, *p* < 0.0001 (20 studies, *n* = 2,374, *I*^2^ = 32%)
					BI	At the end of treatment	MD 15.0, 95% CI 5.83–24.17, *p* = 0.0 (1 study, *n* = 85)
					Death and disability (BI ≤ 60)	3 months	RR 0.71, 95% CI 0.53–0.94; *p* = 0.02 (1 study, *n* = 200)
Cinepazide	Ischemic	Ni, 2020	RCT	937	mRS	90 days	mRS ≤ 2: 284 (60.9%) vs. 236 (50.1%); unadjusted *p* = 0.0004, adjusted for age *p* = 0.001	B-R
							mRS > 2: OR 0.607, 95% CI 0.460–0.801	
					BI	90 days	BI ≥ 95: 53.4 vs. 46.7%; unadjusted *p* = 0.0230, adjusted for age *p* = 0.012
							BI < 95: OR 0.719, 95% CI 0.542–0.956

ANELT, Amsterdam-Nijmegen-Everyday-Language-Test; ANOVA, analysis of variance; ARAT, Action Research Arm Test; BI, Barthel Index; CI, confidence interval; CIAT, constraint induced aphasia therapy; CNS, Canadian Neurological Score; CSS, China Stroke Scale; df, degree of freedom; DTER, Diagnostic Therapeutic Effects of Apoplexy Score; EPO, erythropoietin; ESS, European Stroke Scale; EVT, endovascular thrombectomy; FIM, Functional Independence Measure; FMA, Fugl-Meyer Assessment; GCS, Glasgow Coma Scale; GCSF, granulocyte colony stimulating factor; GOS, Glasgow Outcome Score; GOSE, GOS-extended; HSS, Hemispheric Stroke Scale; ICH, intracerebral hemorrhage; IQR, interquartile range; LD, levodopa; MA, meta-analysis; MD, mean difference; mESSS, modified Edinburgh-Scandinavian Stroke Scale; MPH, Methylphenidate; mRS, modified Rankin Scale; MW, Mann–Whitney; NFDS, Neurological Functional Deficit Score; NIHSS, National Institutes of Health Stroke Scale; n.s., not significant; NSS, Neurological Status Score; OR, odds ratio; PALPA, Psycholinguistic Assessment of Language Processing in Aphasia; RCT, randomized clinical trial; RMI, Rivermead Mobility Index; ROB, risk of bias; RR, risk ratio; SAH, subarachnoid hemorrhage; SEM, standard error of the mean; SIS, Stroke Impact Scale; SMD, standardized mean difference; SR, systematic review; SSS, Scandinavian Stroke Scale; tPA, tissue plasminogen activator; TSS, Toronto Stroke Scale; WAB, Western Aphasia Battery.

**Table 3:** Summary of safety results of included studies.

Treatment	Stroke subtype	Study	Safety
**Antidepressants (selective serotonin reuptake inhibitors)**			
Fluoxetine, sertraline, paroxetine, citalopram, and escitalopram	Ischemic, ICH	Legg, 2021	Death at the end of treatment: RR 1.01, 95% CI 0.82–1.24; *p* = 0.98 (six studies, *n* = 6,090, *I*^2^ = 0%).
			AE at the end of treatment (six studies, *n* = 6,080):
			Seizures: RR 1.40, 95% CI 1.00–1.98; *p* = 0.05, *I*^2^ = 45%
			Bleeding: RR 1.08, 95% CI 0.69–1.70; *p* = 0.73, *I*^2^ = 0%
			Fractures: RR 2.35, 95% 1.62–3.41; *p* < 0.01, *I*^2^ = 0%
**Antidepressants (tri- or tetracyclic)**			
Maprotiline	Ischemic	Dam, 1996	Maprotiline group (*n* = 17): 2 dropped out because they moved, 1 because of seizures, and 2 due to sedation.
Nortriptyline	Ischemic, ICH	Mikami, 2011	Nortriptyline dose adjusted due to blood levels above therapeutic range in 2, sedation 2, and GI symptoms 1.
**Botanicals**			
Di huang yin zi	Ischemic	Yu, 2015	No SAE reported.
			Six in DHYZ group complained of nausea for several days, which disappeared later vs. five in placebo group reported nausea. No patient in both groups left the study because of side effects.
Ginkgo biloba	Ischemic	Chong, 2020	All-cause mortality: RR 1.21, 95% CI 0.29–5.09; *p* = 0.80 (two studies, *n* = 441, *I*^2^ = 43%).
			Extracranial hemorrhage: RR 0.82, 95% CI 0.43–1.57; *p* = 0.55 (five studies, *n* = 547, *I*^2^ = 0%).
			AEs: RR 1.18, 95% CI 0.51–2.71; *p* = 0.70 (six studies, *n* = 794, *I*^2^ = 54%).
			SAE: RR 0.66, 95% CI 0.11–3.84; *p* = 0.64 (two studies, *n* = 430, *I*^2^ = 39%).
			Vascular events: RR 0.73, 95% CI 0.34–1.57; *p* = 0.43 (one study, *n* = 339).
			Cardiovascular events: RR 0.93, 95% CI 0.19–4.52; *p* = 0.92 (one study, *n* = 339).
Ginkgo biloba	Ischemic	Ji, 2020	“Acute stage”: No trial reported all-cause mortality or SAEs. No trial reported recurrence rate outcome. AEs: one trial (*n* = 64) did not report any ADR. One trial (*n* = 106) reported dizziness 1 and nausea 1 on ginkgo vs. dizziness 1 in control group. One trial (*n* = 88) only reported facial flushing (2) on ginkgo. AEs disappeared after symptomatic treatment, one dropped out due to AE. Vascular events: RR 0.70, 95% CI 0.44–1.14; *p* = 0.15 (two studies, *n* = 406, *I*^2^ = 0%).
			“Convalescence stage”: Recurrent rate RR 0.57, 95% CI 0.26–1.25; *p* = 0.16 (two studies, *n* = 396, *I*^2^ = 0%). Mortality RR 1.07, 95% CI 0.41–2.81; *p* = 0.90 (two studies, 396, *I*^2^ = 46%). AEs: one trial (*n* = 64) reported no AE. One trial (*n* = 346) on 6 months of treatment reported vomiting 3, blood sugar change 2, myocardial infarction 1, nephritis 1, sick sinus syndrome 1, and pneumonia 2. Only vomiting is considered related to the study treatment.
MLC601/MLC901	ICH	Kumar, 2020	No SAE was reported. Side effects included a mild case of flushing and a moderate case of lip ulcer. No GI side effects were reported.
MLC601/MLC901	Ischemic	Harandi, 2011	No SAE leading to discontinuation. Common AEs: mild and transient nausea and vomiting in 7 on MLC601. No abnormal changes in blood count or renal and liver function.
MLC601/MLC901	Ischemic	González-Fraile, 2016	Not reported
MLC601/MLC901	Ischemic	Venketasubramanian, 2015	By month 24, rates of death and any vascular or other medical events were similar between study groups. No difference in the rates of renal or hepatic AEs. Neoplasms were reported in 4 in MLC601 group (gynecologic 1, lung 2, and urinary 1) vs. 4 in placebo group (parathyroid 1, lung 2, and urinary 1).
Panax notoginseng	ICH	Xu, 2015	Hematoma volume: 4–7 days MD −0.37, 95% CI −1.60 to 0.87, *p* = 0.56 (six studies, *n* = 648, *I*^2^ = 29%); 10–14 days MD −3.80, 95% CI −5.87 to −1.74, *p* = 0.0003 (three studies, *n* = 332, *I*^2^ = 45%); 20–21 days MD −4.82, 95% CI −8.32 to −1.33, *p* = 0.007 (four studies, *n* = 355, *I*^2^ = 80%); and 28–40 days MD −5.15, 95% CI −5.97 to −4.32, *p* < 0.00001 (four studies, *n* = 437, *I*^2^ = 36%).
			Edema volume: MD 10.78, 95% CI 9.07–12.49, *p* < 0.00001 (three studies, *n* = 274, *I*^2^ = 0%).
			Death: OR 2.78, 95% CI 1.52–5.08, *p* = 0.0009 (seven studies, *n* = 715, *I*^2^ = 0%).
Panax notoginseng	Ischemic	Chen, 2008	AEs: RR 1.30, 95% CI 0.47–3.54; *p* = 0.61 (three studies, *n* = 268).
			Death: RR 0.55, 95% Cl 0.08–3.99, *p* = 0.55 (eight studies, *n* = 660, *I*^2^ = 0%)
**Calcium antagonists**			
Nimodipine, flunarizine, isradipine, nicardipine, fasudil, and lifarizine	Ischemic, ICH	Zhang, 2019	Death at the end of treatment: Overall RR 1.06, 95% CI 0.93–1.20 (22 studies, *n* = 6,323); nimodipine RR 1.02, 95% CI 0.88–1.19 (16 studies, *n* = 5,163), flunarizine RR 1.31, 95% CI 0.94–1.82 (three studies, *n* = 790).
			Death at the end of follow-up: Overall RR 1.07, 95% CI 0.98–1.17 (31 studies, *n* = 7,483); nimodipine RR 1.05, 95% CI 0.96–1.16 (24 studies, *n* = 6,312); flunarizine RR 1.34, 95% CI 1.01–1.77 (three studies, *n* = 790); isradipine RR 1.05, 95% CI 0.60–1.85 (one study, *n* = 234).
			Recurrent stroke at the end of follow-up: Overall RR 0.93, 95% CI 0.56–1.54 (nine studies, *n* = 2,460); nimodipine RR 0.98, 95% CI 0.96–1.11 (six studies, *n* = 1,677); flunarizine RR 0.82, 95% CI 0.35–1.97 (two studies, *n* = 764).
			AEs: Overall RR 1.18, 95% CI 0.81–1.74 (13 studies, *n* = 5,095); nimodipine RR 0.93, 95% CI 0.74–1.16 (11 studies, *n* = 4,604); flunarizine RR 3.16, 95% CI 1.91–5.21 (one study, *n* = 331).
			Hypotension: Overall RR 1.43, 95% CI 0.61–3.38 (six studies, *n* = 1,667); nimodipine RR 1.26, 95% CI 0.50–3.14 (five studies, *n* = 1,648); and others RR 5.50, 95% CI 0.30–101.28 (one study, *n* = 19).
Nimodipine, nicardipine, and magnesium	SAH	Dayyani, 2022	All-cause mortality:
			nimodipine OR 0.73, 95% CI 0.53–1.00; ARR −3.35 95% CI -6.00 to −0.00;
			nicardipine OR 0.87, 95% CI 0.56–1.35;
			magnesium OR 0.93, 95% CI 0.70–1.25.
Magnesium	ICH	Naidech, 2022	Not reported
Magnesium	Ischemic or hemorrhagic	Avgerinos, 2019	Death at 90 days: OR 1.10; 95% CI 0.94–1.29; *p* = 0.24 (five studies, *n* = 4,264, *I*^2^ = 0%).
			AEs meta-analysis not feasible but overall no serious side effects reported.
**Choline nucleotides**			
Citicoline	Ischemic	Martí-Carvajal, 2020	All-cause mortality: RR 0.94, 95% CI 0.83–1.07; *p* = 0.35 (eight studies, *n* = 4,362, *I*^2^ = 0%).
			SAEs: cardiovascular RR 1.04, 95% CI 0.84–1.29 (three studies, *n* = 3,591, *I*^2^ = 0%); CNS RR 1.30, 95% CI 1.07–1.59, *p* = 0.008 (three studies, *n* = 3,591, *I*^2^ = 93%); respiratory RR 1.01, 95% CI 0.78–1.31 (three studies, *n* = 3,591, *I*^2^ = 0%); gastrointestinal RR 0.64, 95% CI 0.40–1.00 (three studies, *n* = 1,370, *I*^2^ = 69%); musculoskeletal RR 1.52, 95% CI 0.50–4.60 (two studies, *n* = 1,293, *I*^2^ = 0%); renal RR 2.04, 95% CI 0.99–4.22 (three studies, *n* = 1,560, *I*^2^ = 0%); hematologic RR 1.27, 95% CI 0.46–3.51 (three studies, *n* = 1,560, *I*^2^ = 0%); severe hepatic RR 1.47, 95% CI 0.48–4.49 (one study, *n* = 267).
			Non-serious AEs: No difference in cardiac disorders, pyrexia, constipation, UTI, headache, nausea and vomiting, agitation, hemorrhagic transformation of stroke, pneumonia, and hypotension (one study, *n* = 2,298).
Citicoline + i.v. tPA or/and endovascular thrombectomy (EVT)	Ischemic	Agarwal 2022	AEs: no significant difference between groups.
			Mortality at 90 days: 5/49 (10.2%) vs. 7/50 (14%), *p* = 0.468.
			Lower respiratory tract infection: 3/49 (6.1%) v. 5/50 (10%).
			Symptomatic ICH: 3/49 (6.1%) vs. 3/50 (6%).
			UTI: 2/49 (4.1%) vs. 3/50 (6%).
**Cholinergics**			
Donepezil	“Stroke”	Berthier, 2006	AEs: donepezil 8 (61%) vs. placebo 3 (23%) (*ꭓ*^2^ = 2.42, *p* = 0.119).
			Donepezil: irritability 4 (30%), insomnia and tiredness 2 (15%), seen only during donepezil titration. Recurrence of poststroke seizures 2 (15%) seen during donepezil maintenance.
			Placebo group: headache 1, abnormal dreams 1, and anorexia 1.
Pre-stroke donepezil, rivastigmine, or galantamine	Ischemic	Wakisaka, 2021	Not reported
**CNS stimulants**			
Amantadine, modafinil	Ischemic, ICH, and SAH	Gagnon, 2020	Amantadine: potential AEs in 5/10 (50%) publications. 5/12 in one study experienced potential AEs, but data were not reported by type of brain injury (TBI, SAH, or encephalitis, stroke). Visual hallucinations are the most common AE across all studies, occurring in 3 (2%). Seizures were observed in 2/12 in one study, both rechallenged with amantadine and seizures did not recur.
			Modafinil: potential AEs evaluated in 7/12 (58%) publications. AEs in one study not broken down by disease state (multiple sclerosis vs. stroke). In 97 modafinil-treated patients, most common AEs were dizziness 5 (5%), dry eyes or mouth 5 (5%), anxiety 4 (4%), and sleep disturbances 4 (4%). No severe AE reported. 14 stopped study drug, nine due to presumed AEs (dizziness 2, anxiety 2, rash 1, and unknown 4).
Amphetamine	Ischemic	Goldstein, 2018	One withdrew consent, one moved away from study site, one transferred to rehabilitation facility closer to home. One with remote history of seizure disorder had a possible uncomplicated partial seizure. One had bilateral lower extremity and deep venous thrombosis (DVT), was found to have colon cancer with hepatic metastases, and died of sepsis in the acute care hospital.
			AEs that did not prompt withdrawal from the study included 1 recurrent stroke 2 months after completing the last study assessment and one DVT treated with an IVC filter. No treatment-associated SAE occurred.
Methylphenidate	Ischemic	Lokk, 2011	No adverse side effects were reported.
**Colony stimulating factors**			
Erythropoietin (EPO), granulocyte colony stimulating factor (GCSF)	Ischemic, ICH	Bath, 2013	Death at the end of trial: EPO OR 1.98, 95% CI 1.17–3.33; *p* = 0.01 (three studies, *n* = 729, *I*^2^ = 0%); GCSF OR 1.3, 95% CI 0.79–2.13; *p* = 0.30 (eight studies, *n* = 546, *I*^2^ = 0%).
			SAE: EPO OR 1.3, 95% CI 0.89–1.9; *p* = 0.18 (one study, *n* = 522); GCSF OR 1.14 95% CI 0.79–1.65; *p* = 0.48 (six studies, *n* = 494, *I*^2^ = 0%).
			Infection: GCSF OR 0.92 95% CI 0.51–1.68; *p* = 0.79 (six studies, *n* = 494, *I*^2^ = 0%).
			WBC count: EPO MD 0.52, 95% CI -0.08–1.12; *p* = 0.09 (one study, *n* = 522); GCSF MD 28.03, 95% CI 23.32–32.73; *p* < 0.0001 (eight studies, *n* = 524, *I*^2^ = 84.77%).
			RBC count: EPO MD 0.17, 95% CI 0.06–0.27; *p* = 0.0 (two studies, *n* = 562, *I*^2^ = 0%); GCSF MD 0.04, 95% CI −0.19–0.28, *p* = 0.71 (three studies, *n* = 94, *I*^2^ = 0%).
			CD34+ GCSF SMD 1.81, 95% CI 1.06–2.57; *p* < 0.0001 (five studies, *n* = 150, *I*^2^ = 64.04%).
EPO, GCSF	Ischemic, ICH	Chen, 2021	All-causes death RR 1.73, 95%CI 0.61–4.92; *p* = 0.735 (*I*^2^ = 0.0%).
			Recurrent stroke RR 0.43, 95% CI 0.14–1.32; *p* = 0.214 (*I*^2^ = 33.1%).
			On active treatment, one study reported headache, bone pain, and transient liver function abnormality; another study reported 1 bone pain 1, DVT 1; another study reported bone pain 6, headache 3; another study reported gastrointestinal reactions 19, bone pain 15, fever 12, and DVT 1.
GCSF	Ischemic, ICH	Huang, 2017	Mortality OR 1.23, 95% CI 0.76–1.97; *p* = 0.40 (*I*^2^ = 0%).
			SAE’s OR 1.11, 95% CI 0.77–1.61; *p* = 0.57 (*I*^2^ = 0%).
EPO + human choriogonadotropin (hCG)	Ischemic	Cramer, 2014	No significant difference in death, SAE, or AE between groups.
			SAE: 12 in 11 patients (placebo 2, low-dose 3, medium-dose 4, and high-dose 3). Most common: cardiac arrest (6) and new stroke (4). None considered possibly, probably, or definitely related to study drug. Rates between groups P > 0·5, two-tailed Fisher’s exact test. 8 fatal (study mortality rate 8·3%: placebo 1, low-dose 1, medium-dose 3, high-dose 3; *p* > 0.5).
			1 asymptomatic DVT on medium-dose, 0 pulmonary embolism or myocardial infarction.
			AE: placebo 52.2%, low-dose 45.8%, medium-dose 79.2%, and high-dose 32.0 (n.s.).
**Dopaminergics**			
Levodopa	Ischemic, ICH	Ford, 2019	132 SAEs in 107 (18%) participants: 74 in 57 (19%) on co-careldopa vs. 58 in 50 (18%) on placebo. Only 2 (3%) on co-careldopa vs. 1 (2%) on placebo suspected to be related to trial medication. No SUSARs reported.
			Deaths: co-careldopa 22/308 (7%) vs. placebo 17/285 (6%).
			Deaths within 8 weeks: co-careldopa 6 (2%) vs. placebo 1 (0%); none considered likely related to study treatment.
			Vomiting: co-careldopa 19 (6%) vs. placebo 9 (3%).
Ropinirole	Ischemic, ICH	Cramer, 2009	Five SAEs, placebo 1 (fall), ropinirole 4 (new ischemic stroke, UTI, facial sensorimotor symptoms, and death from bile duct cancer), deemed unrelated to study medication.
			Non-serious AEs, deemed possibly or probably related to study medication: sleepiness (8 vs. 1), fatigue (6 vs. 0), and dizziness (3 vs.2).
Bromocriptine, pergolide, pramipexole, carbidopa/levodopa, and amantadine	Ischemic, ICH	Conroy, 2005	Not reported.
**Ergots**			
Hydergine	“Stroke”	Bochner, 1973	No AE reported.
**Gamma-aminobutyric acid (GABA) agonists**			
Clomethiazole, diazepam	Ischemic, ICH	Liu, 2018	No significant differences in SAEs in all trials.
			Frequent AEs on chlormethiazole: somnolence and rhinitis. Somnolence: chlormethiazole RR 4.56, 95% CI 3.50–5.95; *p* < 0.0001 (two studies, *n* = 2,527, *I*^2^ = 61.95%). Rhinitis: RR 4.75, 95% CI 2.67–8.46; *p* < 0.0001 (two studies, *n* = 2,527, *I*^2^ = 53.32%).
**Methylxanthines**			
Aminophylline	Ischemic	Bath, 2004a	Death within 4 weeks: OR 1.12, 95% CI 0.49–2.56; *p* = 0.79 (two studies, *n* = 119, *I*^2^ = 0%)
Pentoxifylline, propentofylline	Ischemic	Bath, 2004b	Death within 4 weeks: pentoxifylline OR 0.65, 95% CI 0.41–1.04; *p* = 0.07 (four studies, *n* = 763, *I*^2^ = 64.0%); propentofylline OR 0.49, 95% CI 0.05–5.10; *p* = 0.05 (one study, *n* = 30).
			AEs: two trials found an excess of nausea and vomiting in patients on pentoxifylline.
Theophylline + thrombolysis	Ischemic	Modrau, 2020	Death: theophylline 0, placebo 2. Causes of death: symptomatic ICH and complete infarction of MCA territory.
			Hematoma type I or II at 24 h: theophylline 5 (15%) vs. placebo 6 (19%).
			ICH of any type: theophylline 10 (30%) vs. placebo 8 (26%).
			New stroke within 90 days theophylline 0 vs. placebo 1.
			No statistically significant difference found between groups for any of the safety outcomes.
**Monoamine oxidase (MAO) inhibitors**			
Moclobemide	Ischemic, ICH	Laska, 2005	21 SAEs, all requiring hospitalization: moclobemide 12, placebo 9. None were judged to be drug-related. No death during treatment. Two died during the 6–12-month follow-up period.
			66 AEs reported as drug-related: moclobemide 33, placebo 33.
			10 discontinued moclobemide: 5 depression, 3 anxiety or confusion, 2 refused to continue. 13 discontinued placebo: 8 depression, 1 anxiety, 1 diarrhea, and 3 refused to continue.
Selegiline	Ischemic, ICH	Bartolo, 2015	AEs: selegiline 9, placebo 8—shoulder pain 1 vs. 2, depression 2 vs. 1, gastrointestinal 3 vs. 2. respiratory infection 1 vs. 3, and urinary infection 2 vs. 0.
			No difference in the frequency or pattern of AEs between groups.
**Mood stabilizers**			
lithium	Ischemic	Mohammadianinejad, 2014	High serum level (1.6 mmol/L) 1.
			No SAE. Non-serious AEs attributed to lithium: dry mouth 3, mild transient muscle twitches 1.
**Neuropeptides**			
Cerebrolysin	Ischemic	Ziganshina, 2020	Death at the end of follow-up RR 0.90, 95% CI 0.61–1.32; *p* = 0.58 (six studies, *n* = 1,517, *I*^2^ = 0%).
			SAEs at the end of follow-up RR 1.15, 95% CI 0.81–1.65; *p* = 0.44 (four studies, *n* = 1,435, *I*^2^ = 0%).
			Non-fatal SAEs at the end of follow-up RR 2.15, 85% CI 1.01–4.55; *p* = 0.05 (four studies, 1,435, *I*^2^ = 0%)
Cerebrolysin	Ischemic	Bornstein, 2018	Death OR 0.81, 95% CI 0.50–1.31; *p =* 0.39 (eight studies, *n* = 1869, *I*^2^ = 0%).
			SAEs OR 1.08, 95% CI 0.73–1.59; *p =* 0.70 (seven studies, *n* = 1780, *I*^2^ = 0%).
			AEs OR 1.02, 95% CI 0.83–1.26; *p* = 0.84 (eight studies, *n* = 1880, *I*^2^ = 28.8%).
Cerebrolysin + rehab	Ischemic	Chang, 2016	SAE: cerebrolysin 1 (cholecystitis with gallstone), placebo 1 (hemorrhagic transformation), and none related to study medication.
			Death: 0.
			Vital signs and laboratory values are similar between treatment groups.
Cerebrolysin	SAH	Woo, 2020	Mortality at 30 days (cerebrolysin 0, placebo 3); at 3 months OR 0.46, 95% CI 0.33–0.63; and at 6 months OR 0.46, 95% CI 0.33–0.63.
			SAEs comparable between study groups.
**N-Methyl-D-Aspartate (NMDA) agonists**			
Cycloserine	Ischemic, ICH	Cherry, 2014	No AE reported.
**NMDA antagonists**			
Memantine	Ischemic	Beladi Moghadam, 2021	Four on memantine experienced nausea without vomiting, memantine discontinued.
Memantine + constraint induced aphasia therapy	Ischemic, ICH	Berthier, 2009	No AE documented. One withdrew on placebo due to a seizure episode 1 week after termination of constraint-induced aphasia therapy.
**Norepinephrine/noradrenergics**			
Atomoxetine	Ischemic, ICH	Ward, 2017	Vital signs not significantly different between groups. Mental fatigue, sleepiness, and exhaustion reported in both groups, no clear relatedness to atomoxetine.
Reboxetine	“Stroke”	Zittel, 2007	One patient reported short period of nausea on reboxetine but was able to complete the investigation without impairment. No AE in the placebo group.
**Opioid antagonists**			
Naloxone, nalmefene	Ischemic	Ortiz, 2021	Not reported
Peripheral chemoreceptor agonists			
Almitrine-raubasine	Ischemic	Li, 2004	AEs reported in 3 (8%) on almitrine-raubasine (GPT increase 1, insomnia 2) vs. 1 (3%) on placebo (fibrinogen increase 1), *p* > 0.05. All AEs mild, of short duration and resolved quickly without any treatment.
**Potassium channel blockers**			
Dalfampridine	Ischemic	Page, 2020	Most common treatment-emergent AEs on dalfampridine: fall, urinary tract infection, dizziness, nasopharyngitis, and headache; on placebo: fatigue, nasopharyngitis, fall, arthralgia, pain in extremity, back pain, headache, and hypertension.
			Seizure: dalfampridine 0, placebo 1.
			No hepatic abnormality, no death.
**Pyrazolones**			
Edaravone	ICH	Qin 2022	All-cause mortality: RR 0.51 95% CI 0.11–2.32; *p* = 0.38 (three studies, *n* = 185, *I*^2^ = 0%).
			Hematoma volume (seven studies): MD −4.71 95% CI −5.86 to −3.56; *p* < 0.00001 (seven studies, *n* = 588, *I*^2^ = 88%).
			AE: RR 1.67 95% CI 0.92–3.06; *p* = 0.09 (six studies, *n* = 456, *I*^2^ = 0%).
			24 out of 231 on edaravone developed AE, most frequently reported: kidney impairment, liver impairment, and skin irritation.
Edaravone	Ischemic	Fidalgo, 2022	ICH: OR 0.72, 95% CI 0.44–1.19; *p* = 0.21 (10 studies, *I*^2^ = 78%).
			90-days mortality: OR 0.50, 95% CI 0.45–0.56; *p* = 0.42 (five studies, *n* = 25,129, *I*^2^ = 0%).
Edaravone + i.v. tPA	Ischemic	Hu, 2021	ICH: OR 0.44, 95% CI 0.29–0.66; *p* < 0.00001 (eight studies, *n* = 946, *I*^2^ = 0%)
			Mortality: OR 0.43, 95% CI 0.13–1.42; *p* = 0.87 (four studies, *n* = 442, *I*^2^ = 0%)
Edaravone dexborneol	Ischemic	Xu, 2021	AEs: edaravone-dexborneol 558 (93.16%) vs. edaravone 559 (93.95%).
			SAEs: 54 (9.02%) vs. 47 (7.90%).
			Deaths: 8 (1.34%) vs. 10 (1.68%).
**Racetams**			
Piracetam	Ischemic	Ricci, 2012	Death at 1 month: OR 1.32, 95% CI 0.96–1.82; *p* = 0.09 (three studies, *n* = 1,002, *I*^2^ = 35.37%).
			Death at 12 weeks: OR 1.32; 95% CI 0.97–1.80; *p* = 0.08 (one study, *n* = 927)
Piracetam	“Stroke”	Zhang, 2016	AEs such as gastrointestinal discomfort, anxiety, restlessness or sleep disturbance, vertigo, tiredness, irritability, agitation, and seizures recorded after treatment, but only one trial reported possible treatment-related AE. Six trials reported no AE considered as drug-related, no significant difference in frequency of AEs between groups.
**Vasodilators**			
Buflomedil	Ischemic	Wu, 2015	Death within 3 months: four studies (*n* = 325) reported no death. Four studies (*n* = 731) reported at least one death: RR 0.45, 95% CI 0.14–1.46; *p* = 0.18 (*I*^2^ = 0%).
			AEs in 17 trials, 38 of 955 on buflomedil had AEs, most commonly headache 13 (two stopped) and GI reactions 12. Other AEs: drowsiness 3, pruritus 3, transient dizziness 2, lower blood pressure 2, face flushing 2, and gum bleeding 1. Among 944 controls, two GI reactions.
Cinepazide	Ischemic	Ni, 2020	AE: cinepazide 82% vs. control 84.1%. Most common: constipation (26.0 vs. 26.5%, *p* = 0.82). Hypokalemia: cinepazide 6.1% vs. control 10.5% (*p* = 0.0004). Other AEs did not differ significantly between groups.
			No clinically significant changes in vital signs, most clinical laboratory parameters, and ECG between groups.
			23 deaths (cinepazide 10, control 13), none attributed to study drug. 18 were related to multiple organ dysfunctional syndrome, cerebral hernia, and acute myocardial infarction, five deaths unknown cause.

### Antidepressants, selective serotonin reuptake inhibitor/serotonergic

Eighty-five papers on SSRI in stroke were reviewed. The study selected was a systematic review and meta-analysis that included 13,029 patients from 38 fluoxetine, 13 paroxetine, eight sertraline, nine citalopram, five escitalopram, two citalopram or fluoxetine, and one sertraline or fluoxetine studies (17). Of the six studies at low risk of bias, all on fluoxetine, there was little to no difference in disability, independence, and motor deficit at the end of treatment between groups. When all studies, irrespective of the risk of bias, were included, SSRIs reduced disability scores but not the proportion of independent patients at the end of treatment, except for one study on citalopram (*n* = 642; RR 0.90, 95% CI 0.82–0.98; *p* = 0.01; LOE B-R).

### Antidepressants, tetracyclic or tricyclic

Thirteen papers that investigated tricyclic antidepressants alone or together with other antidepressants in stroke were reviewed. A small RCT (*n* = 46) comparing maprotiline to placebo and fluoxetine in stroke patients who were unable to walk showed that maprotiline is no better than placebo and may hinder recovery in post-stroke patients undergoing rehabilitation (18). In another RCT (*n* = 83) that also compared nortriptyline to placebo and fluoxetine, treatment with anti-depressants, either fluoxetine or nortriptyline, improved modified Rankin Scale (mRS) scores compared to placebo independently of depression [mixed model time–treatment interaction *t*(105) = 2.91, *p* = 0.004; LOE C-LD] (19).

### Botanicals

Fifty-one papers on botanicals were reviewed, of which nine were selected. In a small RCT, 100 patients with ischemic stroke within the prior 30 days were allocated to either di huang yin zi (DHYZ) or placebo for 12 weeks (20). Only 87 patients who completed the study were analyzed, which showed better Fugl-Meyer Assessment (FMA) scores in patients treated with DHYZ at 8 weeks (mean difference, MD, 7.0, 95% CI 1.6–12.4) and 12 weeks (MD 6.5, 95% CI 0.7–12.3) with higher Barthel Index (BI) scores on DHYZ at 12 weeks (MD 4.5, 95% CI 0.3–8.7) (LOE C-LD).

Two systematic reviews on ginkgo biloba, both on patients with ischemic stroke and published in 2020, were selected. One systematic review and meta-analysis included 13 studies (*n* = 1,466) (21). The pooled results suggest that ginkgo biloba was associated with improvement in neurological function on the National Institutes of Health Stroke Scale (NIHSS; MD −2.87, 95% CI −4.01 to −1.74; *p* < 0.00001), in activities of daily living (MD 9.52, 95% CI 4.66–14.38; *p* = 0.00001) and functional outcome (MD −0.50, 95% CI −0.63 to −0.37; *p* < 0.00001) at the end of the study. The second systematic review and meta-analysis included 15 studies (*n* = 1829) (22). Analyses showed that ginkgo biloba improved NIHSS (MD −1.39, 95% CI −2.15 to −0.62; *p* = 0.0004), Neurological Functional Deficit Scores (NFDS, RR 1.20, 95% CI 1.12–1.29; *p* < 0.00001), and activities of daily living (MD 5.72, 95% CI 3.11–8.33; *p* < 0.0001) compared with conventional therapy at different stages after an ischemic stroke. In both reviews, however, many of the studies were judged to be of poor quality and reliability due to the risk of bias (LOE C-LD).

Four studies were selected for MLC601/MLC901. An RCT (*n* = 150) included patients with ischemic stroke within 1 month of onset to either MLC601 or placebo for 3 months (23). Repeated measures analysis showed that motor recovery on FMA was higher in treated patients at 4 weeks (*p* < 0.001), 8 weeks (*p* = 0.001), and 12 weeks (p < 0.001) compared to control (LOE B-R). In a systematic review and meta-analysis of five RCTs (*n* = 1936), pooled analysis on functional recovery at the end of treatment (1 or 3 months) favored MLC601 (RR 1.64, 95% CI 1.05–2.57; *p* = 0.031; LOE B-R) (24). We prioritized the confidence interval in our interpretation of the results rather than the prediction interval calculated by the authors. A long-term follow-up study of patients with ischemic stroke (*n* = 880) showed that the odds ratio (OR) of functional independence was significantly increased at 6 months (1.49, 95% CI 1.11–2.01) and persisted up to 18 months (OR 1.36, 95% CI 1.01–1.83) on mRS and at 6 months (OR 1.55, 95% CI 1.14–2.10) on BI after treatment with MLC601 compared to placebo (LOE B-R) (25). One cohort study (*n* = 66) was the only paper available in patients with ICH (26). While patients treated with MLC601/MLC901 showed a sustained effect on neurological and functional recovery, this was not a controlled study.

Two studies were selected for Panax notoginseng, one each on ischemic stroke and ICH. The systematic review and meta-analysis on ischemic stroke included eight RCTs (n = 660) (27). However, seven of the eight studies were considered to be of poor quality. Pooled analysis (seven RCTs) indicated more improvement in neurological deficit with Panax notoginseng than control (RR 0.29, 95% CI 0.18–0.47, *p* = 0.00001) (LOE C-LD). Meta-analysis of two trials indicated a lower rate of death and dependency at 28 days (RR 0.63, 95% Cl 0.45–0.88; *p* = 0.0072), and one trial reported higher BI on Panax notoginseng (LOE C-LD). The systematic review and meta-analysis of intravenous Panax notoginseng in ICH patients included 20 studies (*n* = 1891), of which four were considered high-quality trials (28). Intravenous Panax notoginseng was associated with better “effectiveness rate” as defined in each study and often calculated as number of cases with desired grade of outcome out of total number in each group (OR 2.70; 95% CI 2.16–3.38; *p* < 0.00001), less neurological deficit (MD 4.36; 95% CI 3.07–5.65; *p* < 0.00001), and increased BI (MD 11.73; 95% CI 19.31–4.16; *p* = 0.002) (LOE B-R).

### Calcium antagonists

One hundred and seven papers on calcium antagonists were reviewed. Four papers were selected. A systematic review and meta-analysis included 34 RCTs (*n* = 7,731) on calcium antagonists in ischemic stroke, of which two studies included hemorrhagic stroke (*n* = 255) (29). The most studied calcium antagonists were nimodipine (26 RCTs) and flunarizine (3 RCTs). No effect on death or disability at the end of follow-up was shown by calcium antagonists as a group or by individual drugs. In a systematic review and network meta-analysis of prophylactic therapies after SAH, improvement in functional outcome at the end of follow-up was seen for nimodipine on Glasgow Outcome Scale (GOS, OR 1.46, 95% CI 1.07–1.99) and nicardipine on mRS (OR 8.80, 95% CI, 1.34–57.77), while magnesium did not reduce mortality or disability despite its effects on delayed cerebral ischemia and vasospasm (LOE A) (30).

A systematic review and meta-analysis of magnesium in ischemic or hemorrhagic stroke within 24 h of onset included seven RCTs (*n* = 4,347) (31). Compared with placebo, magnesium overall improved neither functional outcome (BI >60 or > 95) nor global outcome (mRS) at 90 days post-stroke. A subgroup meta-analysis of three RCTs that exclusively included only ischemic stroke patients (*n* = 164) resulted in lower mRS scores at 90 days post-stroke (weighted mean difference, WMD, −0.96, 95% CI −1.34 to −0.58; *p* < 0.00001), although this should be viewed with extreme caution given the limited number of patients (LOE C-LD). A recent sub-study of a large RCT that investigated the benefit of magnesium administration within 2 h of stroke symptom onset analyzed the subset of patients who suffered an ICH (*n* = 268) (32). In this sub-analysis, magnesium did not improve NIHSS or mRS at 90 days.

### Choline nucleotides

Thirty-six papers were reviewed, and two studies were selected. In a systematic review and meta-analysis of 10 RCTs (*n* = 4,543) on citicoline in patients with ischemic stroke, all were assessed as having a high risk of bias (33). Citicoline did not increase the proportion of patients with NIHSS ≤1 at 6 weeks (4 RCTs) or the proportion of patients with mRS <3 (four RCTs) compared with placebo. Four trials indicated that citicoline did not improve BI, while one study (*n* = 63) showed more patients on citicoline achieving BI scores ≥85 compared to control (RR 3.13, 95% CI 1.10–8.91; *p* = 0.03; LOE C-LD).

A placebo-controlled, blinded endpoint assessment RCT (*n* = 99) investigated the administration of citicoline immediately after recanalization therapy, either intravenous or endovascular, in patients with acute ischemic stroke (34). No differences between treatment groups were seen in neurological (NIHSS) or functional (mRS and BI) outcomes at 90 days.

### Cholinergics

Twenty-two papers were reviewed. Many of the studies were small and assessed cognitive outcomes at endpoints. A small RCT (*n* = 26) on donepezil in chronic post-stroke aphasia was selected (35). Donepezil given for 16 weeks was reported to improve Aphasia Quotient score on the Western Aphasia Battery (*p* = 0.037, Cohen’s *d* = 0.87) and Picture Naming on the Psycholinguistic Assessment of Language Processing in Aphasia (PALPA) (*p* = 0.025, Cohen’s *d* = 0.92), but not in other PALPA subtests, Communicative Activity Log, or the Stroke Aphasic Depression Questionnaire at endpoint (LOE C-LD).

A cohort study on pre-stroke usage of cholinesterase inhibitors (donepezil, galantamine, and rivastigmine) was selected (36). The study analyzed 805 patients with pre-stroke dementia within 7 days of an acute ischemic stroke and followed for 3 months. Patients were stratified according to pre-stroke usage of any cholinesterase inhibitor. Non-usage was associated with neurological deterioration by ≥2 points on NIHSS during hospitalization (OR 0.52, 95% CI 0.31–0.88; *p* = 0.01) and poor functional outcome at 3 months (mRS ≥3 OR 0.68, 95% CI 0.46–0.99; *p* = 0.048) after adjusting for potential confounding factor, as well as after propensity score matching (LOE B-NR).

### Central nervous system stimulants

Thirty papers were reviewed and two studies were selected. Although a systematic review of 11 trials (*n* = 329) on amphetamine was published in 2009 that concluded “no evidence exists at present to support the use of amphetamine after stroke,” and another one on CNS drugs in 2017 (37, 38), a more recently published RCT (*n* = 64) on amphetamine in patients with ischemic stroke and moderate-to-severe motor impairment was selected (39). Amphetamine or placebo was administered 1 h before physiotherapy every 4 days for six sessions. No overall treatment-associated differences in neurological, motor, walking, and functional scores were observed at the end of treatment or at 3 months.

A systematic review of modafinil (*n* = 138 in 12 studies) and amantadine (*n* = 128 in 10 studies) included studies with very varied stroke subtypes (ischemic, ICH, and SAH), some even including other neurological disorders (traumatic brain injury, dementia, etc.) (40). Forty different outcome measures with 141 domains were described across all studies. A positive response in at least one clinical effectiveness measure was reported in 83% of modafinil publications and 70% of amantadine publications. Quantitative analyses were not performed due to heterogeneity in the outcome measures.

Although an RCT (*n* = 21) on methylphenidate published in 2018 was available, it included only patients with post-stroke neglect (41). We, therefore, selected a study (*n* = 78) that compared methylphenidate or/and levodopa with a placebo in patients with a paretic arm and/or leg following a stroke that had occurred 15–180 days before (42). The study found that, compared with a placebo, treatment with methylphenidate, levodopa, or methylphenidate + levodopa combined with physiotherapy improved activities of daily living (ADL, *p* = 0.011) and NIHSS (*p* = 0.001) at 6 months but not motor recovery on FMA (LOE C-LD).

### Colony stimulating factors

Of the 63 papers on colony-stimulating factors, four were selected. In a systematic review and meta-analysis of 11 studies (*n* = 1,275), 3 on EPO and 8 on G-CSF, in patients with ischemic or hemorrhagic stroke, EPO therapy was associated with an increase in death by the end of the trial (OR 1.98, 95% CI 1.17–3.33; *p* = 0.01) with no improvement on neurological impairment or functional outcome. G-CSF was associated with no significant reduction in early impairment and had no effect on functional outcome or death at the end of the trial (43). A more recent systematic review and meta-analysis of eight studies (*n* = 485), one on EPO and seven on G-CSF, also did not show improvement in NIHSS or BI at the end of the follow-up (44). A meta-analysis of G-CSF (*n* = 1,037 in 14 studies) in acute or subacute ischemic or hemorrhagic stroke concluded that while it was associated with a weakly significant improvement on BI (MD 8.65, 95% CI 0.98–16.32; *p* = 0.03), NIHSS was not improved at 3 months (LOE C-LD) (45).

A dose-escalation RCT (*n* = 96) of EPO in combination with human choriogonadotropin (hCG) in patients with supratentorial ischemic stroke was halted early (46). No significant difference in improvement on NIHSS was found between placebo and active treatment, whether analyzed together or separately, as well as on functional outcomes at 90 days.

### Dopaminergics/dopamine agonists

Thirty-six papers on dopaminergics in stroke were reviewed. Although a meta-analysis (six RCTs, *n* = 795) of levodopa in stroke was available in 2020 (47), this was only published in abstract form and was performed in preparation for the Enhancement of Stroke REehabilitation with Levodopa (ESTREL) study. The meta-analysis showed a small non-significant trend for motor recovery in levodopa-treated stroke patients compared to control patients (Standardized Mean Difference, SMD, 0.15, 95% CI -−0.25 to 0.55). Heterogeneity between trials was considerable (*I*^2^ = 67%), and trials differed regarding phases (chronic or acute), dosage and duration of the study treatment, length of follow-up, and outcome measures.

The published results of the Dopamine Augmented Rehabilitation in Stroke (DARS) study, a randomized, double-blind, placebo-controlled trial in 593 patients who cannot walk independently within 5–42 days of stroke, were selected (48). In this study, levodopa did not improve walking after stroke, long-term disability, or functional outcome.

In a small RCT (*n* = 33), ropinirole did not improve gait velocity or motor recovery (49). Moreover, a cohort study suggests that the use of anti-Parkinson’s medications in patients after a stroke may be associated with longer rehabilitation length of stay and poorer functional status compared to those in the entire cohort (50).

No study on apomorphine met the criteria for review. The result of ESTREL is awaited.

### Ergots

Three papers were reviewed. One study (*n* = 57) that randomized ischemic stroke patients to either nicergoline or hydergine (no placebo arm) was analyzed by comparing outcomes before and after treatment in the same patient group rather than between groups (51). A small placebo-controlled RCT (*n* = 21) of oral hydergine for 12 weeks with a *post-hoc* crossover study (*n* = 15) in “convalescing geriatric” stroke patients was, therefore, selected (52). Analyses of both RCT and cross-over phases of this study showed no significant difference between hydergine and placebo on motor functions assessed that included muscle strength testing, hand grip, elbow flexion, walking, and sitting up.

### Gamma-aminobutyric acid agonists

Fourteen studies were reviewed. A systematic review published in 2018 that included four studies on clomethiazole and one study on diazepam (*n* = 3,838) was selected (53). Although there was an indication of potential benefit in the subgroup of patients with total anterior circulation stroke (RR 1.33, 95% CI 1.08–1.63; *p* = 0.01), no benefit was demonstrated overall on neurological impairments or disability for both ischemic and hemorrhagic stroke (LOE B-R).

### GABA antagonists

No study on GABA antagonists, e.g., flumazenil, met the criteria for review and selection.

### Methylxanthines

Eight papers were reviewed, of which three were selected. In the first systematic review and meta-analysis that included two RCTs (*n* = 119) of aminophylline in patients with ischemic stroke, there was no difference in early death and deterioration or death or disability at the end of the follow-up (54). A systematic review and meta-analysis of pentoxifylline and propentofylline, also in ischemic stroke, included five trials (*n* = 793) (55). No study on pentifylline was included. Death or disability was not reduced at the end of the follow-up (two trials). The data for neurological impairment and disability were not in a form suitable for analysis.

More recently, a small RCT (*n* = 64) investigated theophylline as an add-on treatment to thrombolytic therapy in acute ischemic stroke (56). While theophylline as an add-on to thrombolysis improved NIHSS score at 24 h more than thrombolysis alone (MD −3.6, 95% CI −7.1 to −0.1; *p* = 0.043; LOE C-LD), functional independence at 90 days was not different between treatment groups.

### Monoamine oxidase inhibitors

Three papers were reviewed, all relatively small RCTs, and two studies were selected. One RCT (*n* = 89) investigated the administration of moclobemide for 6 months in patients with post-stroke aphasia within 3 weeks of onset (57). Compared to placebo, treatment with moclobemide for 6 months did not enhance the regression of post-stroke aphasia at 6 and 12 months. Another RCT (*n* = 47) allocated patients within 2 weeks of stroke to either selegiline or placebo for 6 weeks in addition to standard rehabilitation (58). While cognitive functioning was improved in the selegiline-treated group, no significant difference in functional recovery on Functional Independence Measure (FIM) was observed at 2 and 6 weeks.

### Mood stabilizers

Four studies were reviewed, and one RCT on lithium (*n* = 66) was selected (59). There were overall no differences between lithium-treated and placebo-treated patients on improvements in the modified NIHSS and hand FMA scores at 30 days, although discrete differences on modified NIHSS (*t*-test *p* = 0.003) and hand FMA (*t*-test *p* = 0.003) in the cortical stroke subgroup (*n* = 27) were observed (LOE C-LD).

### Neuropeptides

Seventy-two papers were reviewed and four papers on cerebrolysin were selected. In a recent systematic review and meta-analysis of seven RCTs (*n* = 1,601) that included patients within 48 h of ischemic stroke onset, cerebrolysin did not reduce all-cause mortality at the end of the follow-up, but may increase non-fatal serious adverse events (RR 2.15, 85% CI 1.01–4.55; *p* = 0.05) (60). There was not enough data to analyze death or dependency. In another earlier systematic review and meta-analysis of nine RCTs (*n* = 1879) that included patients within 72 h of ischemic stroke, more patients improved by ≥4 points on NIHSS at 21 or 30 days on cerebrolysin than placebo (OR 1.60, 95% CI 1.03–2.48; *p* = 0.035). In the meta-analysis of only more severe patients (NIHSS >12 at baseline) from three RCTs, mRS was improved at 90 days (MD 0.39, 95% CI 0.06–0.71; *p* = 0.02) (LOE B-R) (61). Similarly, cerebrolysin combined with rehabilitation in a small RCT (*n* = 66) of patients with subacute ischemic stroke improved FMA in the subgroup of patients with severe motor deficits at baseline (*p* < 0.05) but not in the overall study population (LOE C-LD) (62). A small RCT (*n* = 50) in patients with SAH showed cerebrolysin to be safe and well-tolerated but did not improve the global functional performance of patients even at 6 months (63).

### N-methyl-D-aspartate agonists

Only one study met the PICO criteria for review. In a small (*n* = 20) randomized, double-blind, placebo-controlled trial, cycloserine given 1 h before motor training did not enhance motor learning or motor skill generalization in adults with weakness of upper and lower extremities from stroke (64).

### NMDA antagonists

Nine papers on NMDA antagonists in stroke were reviewed. In a pilot open-label RCT (*n* = 53) of patients with ischemic stroke within 24 h, memantine was associated with improvements in NIHSS during hospitalization (*p* < 0.0001) and BI at 3 months (*p* = 0.002) (LOE C-LD) (65). Patients with chronic post-stroke aphasia (*n* = 28) were randomized in an RCT to memantine or placebo alone for 16 weeks, after that combined with constraint-induced aphasia therapy (CIAT) for 2 weeks, then drug treatment alone for 2 weeks, 4 weeks of washout period, and followed by a 24-week open-label extension study of memantine (66). While memantine or CIAT alone improved aphasia severity, best outcomes on certain aphasia subdomains were achieved by memantine + CIAT at 16 weeks (*p* = 0.002) and 18 weeks (*p* = 0.0001), with the difference between treatment groups persisting on long-term follow-up (LOE C-LD).

No study on dextromethorphan was selected.

### Norepinephrine/noradrenergics

Eight studies were reviewed and two studies were selected. Atomoxetine, paired with 10 sessions of motor training, was investigated in a small pilot RCT (*n* = 12), which showed better recovery on upper limb FMA at the end of the treatment (MD 7.2, 95% CI 1.6–12.7; *p* = 0.016) but not at 1 month or on other motor assessment scales in patients with upper limb weakness ≥6 months after a stroke (LOE C-LD) (67). In another pilot randomized crossover study (*n* = 10), a single dose of reboxetine given before therapy in patients with “chronic” stroke increased tapping speed (ANOVA *p* = 0.048) and grip strength (ANOVA *p* = 0.003) in the paretic but not in the unaffected hand, with no further improvement noticed after physiotherapy alone (LOE C-LD) (68).

### Opioid antagonists

Eight studies of opioid antagonists in stroke were reviewed. A systematic review published in 2021 that included four studies on naloxone (*n* = 96) and three studies on nalmefene (*n* = 916) was selected (69). From this review, one small study (*n* = 44) on naloxone showed benefit on Neurological Status Score at 2 weeks (*p* < 0.01) and one study (*n* = 236) on nalmefene showed improvement on Glasgow Coma Scale (GCS) at 10 days (*p* < 0.05) and NIHSS at 20 days (*p* < 0.05) compared to controls (LOE C-LD). Meta-analysis was not performed because of the different parameters used in all studies.

### Peripheral chemoreceptor agonists

Two papers were reviewed. A small placebo-controlled RCT (*n* = 74) of almitrine-raubasine in patients with non-acute ischemic stroke was selected (70). Almitrine-raubasine was associated with better BI scores at 1 month (*t*-test *p* = 0.001), 2 months (*t*-test *p* = 0.002), and 3 months (*t*-test *p* = 0.002), and mean improvement on NFDS (*t*-test *p* = 0.034) at 1 month (LOE C-LD).

### Potassium channel blockers

Two papers, both on dalfampridine, were reviewed. The paper selected was an RCT (*n* = 377) that compared two doses of dalfampridine administered for 12 weeks to placebo in patients with walking deficits ≥6 months after an ischemic stroke (71). Dalfampridine, at either 7.5 or 10 mg dose, did not significantly increase walking performance at the end of treatment, although the study was terminated early before the full enrolment of 540 subjects due to an unblinded analysis that showed insufficient efficacy to support further recruitment.

### Pyrazolones

Seventy-two papers, all on edaravone, were reviewed. In a systematic review and meta-analysis of 50,536 patients with ischemic stroke from 14 observational studies and five RCTs, edaravone treatment was overall associated with improved odds of excellent (mRS ≤1 OR 1.26, 95% CI 1.04–1.54; *p* = 0.02) and good (mRS ≤2 OR 1.31, 95% CI 1.03–1.67; *p* = 0.03) functional outcomes with lower mortality. However, study heterogeneity was high, and the effect was reduced to *p* > 0.05 when analysis was restricted to randomized trials only (72). A systematic review and meta-analysis of 17 RCTs on edaravone in patients with ischemic stroke treated with intravenous thrombolysis (*n* = 1877) showed that combined treatment with edaravone and alteplase reduced the NIHSS score at the end of treatment (MD 3.95, 95% CI 2.92–4.99; *p* < 0.00001), 7 days (MD 5.11, 95% CI 2.84–7.37; *p* < 0.00001), and 14 days (MD 3.11, 95% CI 2.23–3.99; *p* < 0.00001) compared with alteplase alone, with less occurrence of intracranial hemorrhage during hospitalization (LOE B-R) (73). A recent RCT (*n* = 1,194) compared edaravone alone with the combination of edaravone and dexborneol in patients with ischemic stroke within 48 h of onset. Improvement on NIHSS at 14 days (MD −0.40, 95% CI −0.72 to −0.08; *p* = 0.01) and mRS at 3 months (OR 1.42, 95% CI 1.12–1.81; *p* = 0.004) were better with combination treatment than edaravone alone (LOE B-R) (74).

A systematic review and meta-analysis of 38 RCTs on edaravone initiated within 7 days of ICH (*n* = 3,453) showed alleviation of neurological deficits (MD −5.44 95% CI −6.44 to −4.44; *p* < 0.00001), improved activities of daily living (MD 8.44 95% CI 7.65–9.23; *p* < 0.00001), and reduced hematoma volume with edaravone, although the included trials were of poor quality and high heterogeneity (LOE C-LD). No studies reported long-term functional outcomes (75).

### Racetams

Twenty-three papers were reviewed and two papers on piracetam were selected. In a systematic review and meta-analysis of three trials (*n* = 1,002) in acute ischemic stroke, most data came from one large trial and overall did not demonstrate the superiority of piracetam over control in improving functional outcome (BI) or reducing death or dependency at 3 months (76). Its role in post-stroke aphasia was investigated in a systematic review and meta-analysis of seven RCTs (*n* = 261), which showed that piracetam did not improve overall language performance but may benefit written language ability (SMD 0.35, 95% CI 0.04–0.66; *p* = 0.03), particularly more so during the first 12 weeks but not long term (LOE C-LD) (77).

### Vasodilators

Two papers were reviewed and selected. In a systematic review and meta-analysis of 26 RCTs on buflomedil in patients treated within the first few days of ischemic stroke (*n* = 2,756), the trials were generally of poor quality, and many were poorly reported (78). Only one trial (*n* = 200) reported long-term death and disability, with patients on buflomedil having a lower risk of death or disability than the control group at 3 months (RR 0.71, 95% CI 0.53–0.94; *p* = 0.02). Another trial (*n* = 85) reported less disability (MD 15.0, 95% CI 5.83–24.17, *p* = 0.0) while all 26 trials (*n* = 2,756) reported improvements in neurological deficits at the end of treatment on buflomedil, although evidence for any of these short-term outcomes was not considered robust (LOE C-LD).

A recent RCT (*n* = 937) compared cinepazide to placebo in patients with ischemic stroke within 48 h of onset (79). The study showed cinepazide injection to be safe and better than placebo in improving functional recovery (OR 0.607, 95% CI 0.460–0.801) and reducing disability (OR 0.719, 95% CI 0.542–0.956) at 3 months (LOE B-R).

## Discussion

We performed this systematic-search-and-review to identify the best available evidence of different registered pharmacological interventions for improving recovery after a stroke. Among the different pharmacological interventions reviewed, only one intervention, nimodipine in SAH, was shown to have level A evidence of treatment benefit based on a systematic review and network meta-analysis. Other treatments with LOE A studies did not demonstrate the benefit of intervention over control, namely SSRIs, calcium antagonists, and citicoline in ischemic stroke and magnesium, colony-stimulating factors, and GABA agonists in ischemic or hemorrhagic stroke.

Many of the reviewed selected papers were assessed as LOE C-LD, mostly due to small sample sizes or poor quality of studies, some of which showed treatment effects and required larger studies to provide better certainty of evidence. Of the studies assessed as LOE B-R, seven commercially available drugs showed treatment effects, although additional trials would further support their clinical use. While generally reported to be safe, it is important as well to be aware that certain treatments may have a detrimental effect on poststroke recovery (80). In our review, the risk may be increased for bone fracture on SSRI (17), non-fatal serious adverse events on cerebrolysin (60), and death at the end of the study on flunarizine and EPO (29, 43).

The studies of different interventions in our review included patients with a wide range of treatment time windows from stroke onset. As the underlying pathophysiological targets after an ischemic stroke differ between the time of injury and during repair (81–83), we may arbitrarily consider interventions given within 24 or 48 h as “neuroprotective,” of which re-establishment of blood flow and reperfusion to the injured brain tissue is currently the best strategy, and those administered beyond 48 h as “recovery-promoting” treatments. Recovery-promoting therapies should be viewed separately from those that enhance neuroprotection or reperfusion after a stroke since they have distinct therapeutic targets that are related to plasticity and growth after stroke with a therapeutic time window measured in days, weeks, or even months that may benefit a larger proportion of patients with stroke (84).

In our review, level B-R evidence of treatment effect at 3 months was available only for edaravone (particularly when combined with dexborneol) and cinepazide when administered within 48 h and clomethiazole (in total anterior circulation syndrome) within 12 h of acute ischemic stroke (53, 72, 74, 79). More recently, re-evaluating drugs as adjunctive therapies to revascularization have gained interest since many of these compounds were investigated when thrombolysis and endovascular thrombectomy were rarely available (85–88). Furthermore, despite the higher recanalization rate and efficacy of thrombectomy, approximately half of patients still had poor outcomes at 90 days (89). Our review identified four drugs recently tested in combination with revascularization attempts—citicoline, clomethiazole (included in the systematic review on GABA agonists), theophylline, and edaravone (34, 53, 56, 73). Some results are promising and such approach may be important to consider when designing future trials to re-assess supposed neuroprotective drugs, especially when taken in the context of learnings from recent studies of novel compounds given to patients receiving reperfusion therapies (90–92).

Among interventions administered beyond 48 h of stroke onset, level B-R evidence of therapeutic effect was available for SSRIs (at the end of treatment), MLC601/MLC901 (at 3 to 18 months), and cerebrolysin (at 1 or 3 months) in ischemic stroke, and Panax notoginseng (at 1 month) in ICH (17, 23–25, 28, 61). Our review also revealed investigations of other multi-modal approaches that included combination treatments, i.e., botanicals and EPO + hCG, and administering treatment together with or before planned rehabilitative training, particularly for potential recovery-promoting drugs. Combining different therapy principles is a logical step to further increase poststroke recovery, wherein a simplified theoretical scheme uses priming treatments synergistically with respective consolidation treatments (training) (93, 94). Further research on putative recovery-promoting treatments can benefit much from new approaches to patient selection, inclusion of more severe deficits, control interventions, appropriate outcome measures based on the intervention’s target and stage of stroke recovery, and longer duration of follow-up (95, 96).

Our systematic-search-and-review have several limitations. We did not include a standardized quality assessment tool in reviewing each of the papers as this is not required for a systematic-search-and-review study design especially aimed at exhaustive searches. Because of the broad range of treatments covered, we did not do any meta-analysis to obtain pooled results from different studies. This was, however, not the objective of the review and can be performed as a next step focusing on selected pharmacological classes or products. We included only papers published in the English language and may have excluded some well-conducted large studies published in other languages. One drug in particular, cortexin, had all studies only in non-English publications and was therefore not reviewed. As mentioned in the Methods section, we excluded studies that assessed psychiatric and cognitive outcomes, as well as spasticity, seizures, pain, and fatigue. We also excluded imaging and laboratory outcomes as surrogate markers since we were mainly interested in post-stroke clinical outcomes as hard endpoints. In addition, our review did not include studies on less usual causes of stroke, e.g., stroke in pregnancy and stroke in children. Finally, we focused mainly on treatments that clinicians would have ready access to rather than on investigational new drugs because we intended this review to guide clinical decision-making.

In conclusion, only one registered treatment has level A evidence for routine use in patients who suffer an acute stroke—nimodipine after SAH. There are, however, several commercially available treatments with level B evidence as either neuroprotective or recovery-promoting treatments. Further studies of putative neuroprotective drugs as adjunctive treatment to revascularization procedure, as well as more confirmatory studies on neuro-recovery treatments, will enhance the certainty of their benefit seen in clinical trials. As most molecular targets for therapy have biphasic roles in stroke pathophysiology during acute injury and in neurovascular remodeling in the recovery phase (82, 97), an intervention that failed as a neuroprotectant may not necessarily be of no benefit as a recovery-promoting treatment after a stroke. Even treatments with level C evidence may be candidates for larger studies, particularly those with signals on preclinical and clinical studies. Study designs must be based on the expected mechanism of action and stroke subtype and aimed at restoring the specific impairment at the optimal time window. Moreover, treatment for neuro-recovery may require a much longer duration than neuroprotective trials.

As the treatments we reviewed are registered products and may be available to clinicians and patients, the decision on their use must be guided by the clinical profile, neurological impairments, and outcomes they hope to improve based on the available evidence outlined.

## Data availability statement

The original contributions presented in the study are included in the article/Supplementary material; further inquiries can be directed to the corresponding author.

## Author contributions

T-HL: Conceptualization, Formal analysis, Investigation, Methodology, Project administration, Resources, Supervision, Validation, Writing – original draft, Writing – review & editing. SU: Formal analysis, Investigation, Methodology, Supervision, Validation, Writing – review & editing. YK: Formal analysis, Investigation, Validation, Writing – review & editing. HCC: Formal analysis, Investigation, Validation, Writing – review & editing. JCN: Formal analysis, Investigation, Validation, Writing – review & editing. KST: Formal analysis, Investigation, Validation, Writing – review & editing. JP: Formal analysis, Investigation, Validation, Writing – review & editing. LG: Data curation, Methodology, Project administration, Software, Supervision, Writing – review & editing. YW: Data curation, Methodology, Project administration, Software, Supervision, Writing – review & editing. NV: Conceptualization, Formal analysis, Investigation, Methodology, Project administration, Supervision, Validation, Writing – original draft, Writing – review & editing.

## Footnotes

^1^ https://www.covidence.org
