# Supplementary material for: A systematic-search-and-review of registered pharmacological therapies investigated to improve neuro-recovery after a stroke
Source: Front Neurol. 2024 Jan 31;15:1346177. doi: 10.3389/fneur.2024.1346177 (PMC10866005; doi:10.3389/fneur.2024.1346177)
Supplement: Supplementary file 1 [file Data_Sheet_1.pdf]

SUPPLEMENTS

Table S1. Pharmacological classes and products in the systematic search and review

Antidepressants, selective serotonin reuptake inhibitor (SSRI)/serotonergic
- Citalopram
- Escitalopram
- Fluoxetine
- Paroxetine
- Sertraline

Antidepressants, tetracyclic or tricyclic
- Maprotiline
- Nortriptyline

Botanicals
- Di Huang Yin Zi
- Gingko biloba
- MLC601/MLC901
- Panax notoginseng

Calcium antagonists
- Nimodipine
- Magnesium

Choline nucleotides
- Citicoline

Cholinergics
- Donepezil

CNS stimulants
- Amphetamine/dextro-amphetamine
- Methylphenidate
- Modafinil

Colony stimulating factors
- Erythropoietin (EPO)
- Granulocyte colony stimulating factor (G-CSF)/filgrastim

Dopaminergics / dopamine agonists
- Levodopa
- Ropinirole
- Apomorphine

Ergots
- Hydergine
- Nicergoline

Gamma-aminobutyric acid (GABA) agonists
- Diazepam
- Chlormethiazole

GABA antagonists
- Flumazenil

Methylxanthines
- Theophylline

Monoamine Oxidase (MAO) inhibitors
- Selegiline
- Moclobemide

Mood stabilizers
- Lithium

Neuropeptides
- Cerebrolysin
- Cortexin

N-Methyl-D-aspartate (NMDA) agonists
- Cycloserine

NMDA antagonists
- Dextromethorphan
- Memantine

Norepinephrine / Noradrenergics
- Reboxetine
- Atomoxetine
- Atipamezole

Opioid antagonists
- Naloxone
- Naltrexone
- Nalmefene

Peripheral chemoreceptor agonists
- Almitrine-Raubasine

Potassium channel blockers
- Dalfampridine

Pyrazolones
- Edaravone

Racetams
- Piracetam

Vasodilators
- Buflomedil
- Cinepazide

Table S2. Search Strategy in EMBASE

#1	'stroke'/exp/mj OR stroke
#2	recover* OR prognos* OR function* OR 'motor'/exp OR motor OR neuro*
#3	'treatment outcome'/exp/mj OR ('treatment' AND 'outcome')
#4	#2 AND #3
#5	'drug therapy'/exp/mj OR ('drug' AND ('therapy' OR 'treatment' OR 'prescription'))
#6	('pharmacology'/exp/mj OR 'pharmacology' OR pharmacolog*) AND product*
#7	#5 OR #6
#8	'antidepressant'/exp/mj OR antidepressant OR tetracyclic OR tricyclic OR 'maprotiline'/exp/mj OR maprotiline OR 'nortriptyline'/exp/mj OR nortriptyline
#9	'selective serotonin reuptake inhibitor'
#10	'ssri'/exp/mj OR ssri OR serotonergic OR 'citalopram'/exp/mj OR citalopram OR 'escitalopram'/exp/mj OR escitalopram OR 'fluoxetine'/exp/mj OR fluoxetine OR 'paroxetine'/exp/mj OR paroxetine OR 'sertraline'/exp/mj OR sertraline
#11	'mood stabilizer'
#12	'lithium'/exp/mj OR 'lithium'
#13	'norepinephrine'/exp/mj OR norepinephrine OR noradrenergic OR 'reboxetine'/exp/mj OR reboxetine OR 'atomoxetine'/exp/mj OR atomoxetine OR 'atipamezole'/exp/mj OR atipamezole
#14	'n-methyl-d-aspartate antagonist'/exp/mj OR 'n-methyl-d-aspartate antagonist'
#15	'dextromethorphan'/exp/mj OR dextromethorphan OR 'memantine'/exp/mj OR memantine
#16	'calcium antagonist'/exp/mj OR 'calcium antagonist' OR 'nimodipine'/exp/mj OR nimodipine
**#17**	botanical OR 'di huang yin zi' OR 'gingko biloba'/exp/mj OR 'gingko biloba' OR 'mlc601' OR 'mlc901'
#18	('monoamine oxidase'/exp/mj OR 'monoamine oxidase' OR 'mao'/exp/mj OR mao) AND ('inhibitor'/exp/mj OR inhibitor)
#19	'cholinergic'/exp/mj OR cholinergic OR 'donepezil'/exp/mj OR donepezil
#20	'choline nucleotide' OR 'citicoline'/exp/mj OR citicoline

#21	'peripheral chemoreceptor agonist' OR 'almitrine raubasine'/exp/mj OR 'almitrine raubasine'
#22	'methylxanthine'/exp/mj OR methylxanthine OR 'theophylline'/exp/mj OR theophylline OR 'erythropoietin'/exp/mj OR erythropoietin OR 'ergot'/exp/mj OR ergot OR 'hydergine'/exp/mj OR hydergine
#23	('central nervous system'/exp/mj OR 'central nervous system' OR 'cns'/exp/mj OR cns) AND ('stimulant'/exp/mj OR stimulant)
#24	'amphetamines'/exp/mj OR amphetamines OR 'dextro amphetamine'/exp/mj OR 'dextro amphetamine' OR 'methylphenidate'/exp/mj OR methylphenidate
#25	'opioid antagonist'/exp/mj OR 'opioid antagonist' OR 'naloxone'/exp/mj OR naloxone OR 'naltrexone'/exp/mj OR naltrexone
#26	'n-methyl-d-aspartate agonist'/exp/mj OR 'n-methyl-d-aspartate agonist' OR 'cycloserine'/exp/mj OR cycloserine
#27	'flumazenil'/exp/mj OR flumazenil
#28	('gamma-aminobutyric acid'/exp/mj OR 'gamma-aminobutyric acid' OR 'gaba'/exp/mj OR gaba) AND ('agonist'/exp/mj OR agonist)
#29	'diazepam'/exp/mj OR diazepam OR 'chlormethiazole'/exp/mj OR chlormethiazole
#30	(dopaminergic OR 'dopamine'/exp/mj OR dopamine) AND ('agonist'/exp/mj OR agonist)
#31	'levodopa'/exp/mj OR levodopa OR 'ropinirole'/exp/mj OR ropinirole OR 'apomorphine'/exp/mj OR apomorphine
#32	racetams OR 'piracetam'/exp/mj OR piracetam OR 'pyrazolone'/exp/mj OR pyrazolone OR 'edaravone'/exp/mj OR edaravone OR 'granulocyte colony stimulating factor'/exp/mj OR 'granulocyte colony stimulating factor' OR gcsf OR 'g csf'/exp/mj OR 'g csf' OR 'filgrastim'/exp/mj OR filgrastim OR 'neuropeptide'/exp/mj OR neuropeptide OR 'cerebrolysin'/exp/mj OR cerebrolysin OR 'cortexin'/exp/mj OR cortexin
#33	#8 OR #9 OR #10 OR #11 OR #12 OR #13 OR #14 OR #15 OR #16 OR #17 OR #18 OR #19 OR #20 OR #21 OR #22 OR #23 OR #24 OR #25 OR #26 OR #27 OR #28 OR #29 OR #30 OR #31 OR #32
#34	#7 AND #32
#35	#1 AND #4 AND #34

#36	#35 AND ('case control study'/de OR 'case report'/de OR 'clinical trial'/de OR 'cohort analysis'/de OR 'comparative study'/de OR 'controlled clinical trial'/de OR 'controlled study'/de OR 'drug dose comparison'/de OR 'evidence based medicine'/de OR 'human'/de OR 'intervention study'/de OR 'major clinical study'/de OR 'meta analysis'/de OR 'multicenter study'/de OR 'observational study'/de OR 'outcomes research'/de OR 'parallel design'/de OR 'phase 2 clinical trial'/de OR 'phase 3 clinical trial'/de OR 'practice guideline'/de OR 'prospective study'/de OR 'randomized controlled trial'/de OR 'randomized controlled trial topic'/de OR 'retrospective study'/de OR 'systematic review'/de)

Table S3. The characteristics of the 58 included studies.

Study	Treatment	Study design	Study N	Blinding	Dose, frequency, route	Treatment duration	Treatment N	Comparator	Comparator N	Age criteria	Treatment window	Stroke severity, impairments	Stroke subtype	Other key criteria	Age of included	Sex (M / F)
Antidepressants (selective serotonin reuptake inhibitors)																
Legg 2021	fluoxetine, sertraline, paroxetine, citalopram, escitalopram	SR/MA	13029 (76 RCTs)		any	any		Placebo or usual care			<12 months		Ischemic, ICH		means from 51±7 years to 75.6 years	M > F
Antidepressants (tri- or tetracyclic)																
Dam 1996	maprotiline	RCT	46	Assessor Blind	150 mg daily, oral	3 months	14	Placebo; fluoxetine 20 mg/d	16; 16		1 to 6 months	unable to walk	Ischemic	MCA territory	mean 68.0±7.3 years	20 (43.5%) / 26 (56.5%)
Mikami 2011	nortriptyline	RCT	83 (depressed 46, non-depressed 37)	Double Blind	25 mg titrated up to 100 mg daily, oral	12 weeks	22	Placebo; fluoxetine 10 mg to 40 mg daily	29; 32	18 to 85 years	<6 months		Ischemic, ICH		mean 65.7±12.4 vs. 72.5±9.4 years	51 (61.4%) / 32 (38.6%)
Botanicals																
Yu 2015	di huang yin zi	RCT	87	Double Blind	18 gm b.i.d., oral	12 weeks	45	Placebo	42	40 to 72 years	<30 days		Ischemic	anterior cerebral circulation	mean 60.7±12.2 vs. 58.1±10.7 years	58 (66.7%) / 29 (33.3%)
Chong 2020	gingko biloba	SR/MA	1466 (13 RCTs)		120 to 160 mg daily, oral or i.v.	4 weeks to 6 months (oral) or 2 to 3 weeks (i.v.)		Placebo, usual care or active control		any	any	any	Ischemic		means from 40 to 70 years	F: from 24.2% to 62.1%
Ji 2020	gingko biloba	SR/MA	1829 (15 RCTs)		any	≥2 weeks	926	Placebo or conventio	903	any	any		Ischemic		mean from 57.38 to 75.2 years	

Study	Treatment	Study design	Study N	Blinding	Dose, frequency, route	Treatment duration	Treatment N	Comparator	Comparator N	Age criteria	Treatment window	Stroke severity, impairments	Stroke subtype	Other key criteria	Age of included	Sex (M / F)
								nal treatment								
Harandi 2011	MLC601/ MLC901	RCT	150	Double blind	4 caps (1.6 g) t.i.d., oral	3 months	100	Placebo	50	30 to 72 years	<1 month		Ischemic		mean 64.41±5.89 vs. 66.14±5.36 years	72 (48%) / 78 (52%)
González-Fraile 2016	MLC601/ MLC901	SR/MA	1936 (5 RCTs)		4 caps (1.6 g) t.i.d., oral	4 to 12 weeks	1065	Placebo or active	871				Ischemic			
Venketasubramanian 2015	MLC601/ MLC901	RCT	880	Double Blind	4 caps (1.6 g) t.i.d., oral	3 months	446	Placebo	434	≥18 years	<72 hours	NIHSS 6 to 14	Ischemic		mean 61.8±11.3 years	562 (63.9%) / 318 (36.1%)
Kumar 2020	MLC601/ MLC901	Cohort study	66	None	2 caps (800 mg) MLC901 or 4 caps (1.6 g) MLC601 t.i.d., oral	3 months	66	None	0	any			ICH		median 58.5 (range 28 to 87) years	44 (67%) / 22 (33%)
Chen 2008	panax notoginseng	SR/MA	660 (8 RCTs)	Double blind, single blind, unknown	any	any		Placebo or "not treated"		any	<30 days		Ischemic		range 47 to 81 years	M > F
Xu 2015	panax notoginseng	SR/MA	1891 (20 RCTs)		any, i.v.	10 to 70 days	984	Current treatment	907				ICH		range 24 to 92 years	62% / 38%
<u>Calcium antagonists</u>																
Zhang 2019	nimodipine, flunarizine, isradipine, nicardipine,	SR/MA	7731 (34 RCTs, 2 studies included 225 ICH)		any, oral or i.v.	any		Placebo or usual care			<14 days		Ischemic, ICH		means from 52.3 to 74.6 years	M > F

Study	Treatment	Study design	Study N	Blinding	Dose, frequency, route	Treatment duration	Treatment N	Comparator	Comparator N	Age criteria	Treatment window	Stroke severity, impairments	Stroke subtype	Other key criteria	Age of included	Sex (M / F)
	fasudil, lifarizine															
Dayyani 2022	nimodipine, nicardipine, magnesium	SR/MA	5234 (25 studies: nimodipine 10, nicardipine 2, magnesium 13) of 10415 (53 network studies)		any	any		Placebo or not treated or active control					SAH		means from 43.5 to 60 years	F: from 25.5 to 86%
Avgerinos 2019	magnesium	SR/MA	4347 (7 RCTs)	Double-blind	any, i.v.	any	2184	Placebo	2163	>18 years	>15 min and <24 hours		Ischemic, ICH		means from 62.5 to 75 years	M > F
Naidech 2022	magnesium	Sub-analysis of RCT	268	Double blind	4 g LD then 16 g over 24h, i.v.	once	145	Placebo	123	40 to 95 years	<2 hours	motor deficit on modified Los Angeles Prehospital Stroke Screen	ICH	Had repeat scan done <24h of arrival	mean 65.4±13.4 years	179 (66.8%) / 89 (33.2%)
<u>Choline nucleotides</u>																
Martí-Carvajal 2020	citicoline	SR/MA	4543 (10 RCTs)		any	any		Placebo, usual care or other intervention		children or adult			Ischemic		mean 69.27±2.05 vs. 70.54±2.02 years (6 RCTs)	M: 57.34% vs. 50.28% (8 RCTs)
Agarwal 2022	citicoline + i.v. tPA or/and endovascula	RCT	99	Assessor Blinded	1 gm b.i.d., i.v. for 3 days then oral	6 weeks	49	Placebo i.v. then oral	50	≥18 years			Ischemic	receive either or both i.v.	median 61±14.5 vs. 54.5±14.6 years	60 (60.6%) / 39 (39.4%)

Study	Treatment	Study design	Study N	Blinding	Dose, frequency, route	Treatment duration	Treatment N	Comparator	Comparator N	Age criteria	Treatment window	Stroke severity, impairments	Stroke subtype	Other key criteria	Age of included	Sex (M / F)
	r thrombectomy (EVT)							multivitamins						tPA or EVT		
Cholinergics																
Berthier 2006	Donepezil	RCT	26	Double blind	5 to 10 mg daily, oral	16 weeks	13	Placebo	13	<70 years	≥1 year	chronic aphasia	"post-stroke"		mean 48.1±9.7 years	18 (69.2%) / 8 (30.8%)
Wakisaka 2021	pre-stroke donepezil, rivastigmine, or galantamine	Cohort study	805	None	any	any	212	"Not treated" with cholinesterase inhibitor pre-stroke	593 (212 propensity score matched)		included in registry <7 days		Ischemic	pre-stroke dementia	mean 83.2±6.5 vs. 81.4±8.3 years	388 (48.2%) / 417 (51.8%)
Central nervous system stimulants																
Gagnon 2020	amantadine, modafinil	SR	amantadine n=128 (10 studies), modafinil n=138 (12 studies)		amantadine 100 mg to 400 mg daily, oral; modafinil 25 to 400 mg daily, oral	6 weeks to 18 months	121, 120	Control or none		≥18 years			Ischemic, ICH, SAH			
Goldstein 2018	amphetamine	RCT	64	Double blind	10 mg 1h prior to 1h PT every 4 days, oral	6 sessions	32	Placebo	32		10 to 30 days	motor impairment (FMA <80)	Ischemic	hemispheric	median 66 (range 27 to 91) years	35 (54.7%) / 29 (45.3%)
Lokk 2011	Methylphenidate (MPH)	RCT	78	Double blind	MPH 20 mg ± levodopa (LD) 125 mg, 60 min before training, oral	5 weeks, 15 sessions	MPH 19, LD 20, MPH+LD 19	Placebo	20		15 to 180 days	paretic arm and/or leg	Ischemic		mean 64±9.8 years	48 (61.5%) / 30 (38.5%)

Study	Treatment	Study design	Study N	Blinding	Dose, frequency, route	Treatment duration	Treatment N	Comparator	Comparator N	Age criteria	Treatment window	Stroke severity, impairments	Stroke subtype	Other key criteria	Age of included	Sex (M / F)
<u>Colony stimulating factors</u>																
Bath 2013	erythropoietin (EPO), granulocyte colony stimulating factor (GCSF)	SR/MA	1275 (11 RCTs: EPO 3, GCSF 8)	double blind 8, single blind 1, unblinded 2							<30 days		Ischemic, ICH			
Chen 2021	EPO, GCSF	SR/MA	485 (8 RCTs: EPO 1, GCSF 7)							adults			Ischemic, ICH	subacute or chronic	mean from 46.4 to 75.3 years	M: from 32% to 68.3%
Huang 2017	GCSF	SR/MA	1037 (14 RCTs)					Placebo or no intervention					Ischemic, ICH	acute or subacute		
Cramer 2014	EPO + human choriogonadotropin (hCG)	RCT dose-escalation study	96	Double Blind	hCG 10,000 IU on days 1, 3, 5, s.c.; then EPO 4,000 IU, 12,000 IU, or 20,000 IU) on days 7, 8, 9, i.v.	9 days	72 (24, 24, 24)	Placebo	24	18 to 85 years	24 to 48 hours	NIHSS 8 to 20	Ischemic	supratentorial	mean 56.9±12.3 vs. 61.7±12.4 years	31 (32.3%) / 65 (67.7%)
<u>Dopaminergics</u>																
Ford 2019	Levodopa	RCT	593	Double blind	Levodopa/carbidopa 50/12.5 mg for 2 doses then 100/25 mg, 45 to 60 min before	6 weeks	308	Placebo	285	≥18 years	5 to 42 days	Could not walk independently ≥10 m	Ischemic, ICH	Rivermead Mobility Index (RMI) <7	mean 67.5±13.6 vs. 69.6±12.7 years	364 (61.4%) / 229 (38.6%)

Study	Treatment	Study design	Study N	Blinding	Dose, frequency, route	Treatment duration	Treatment N	Comparator	Comparator N	Age criteria	Treatment window	Stroke severity, impairments	Stroke subtype	Other key criteria	Age of included	Sex (M / F)
					PT or OT, oral											
Cramer 2009	ropinirole	RCT	33	Double blind	0.25 mg titrated up weekly to 4 mg daily, oral	9 weeks	17	Placebo	16	18 to 80 years	1 to 12 months	Motor deficits (FMA 23 to 83/100); moderate gait difficulty (50-ft walk ≥15 sec or FMA ambulation score >3)	Ischemic, ICH		mean 63±13 vs. 60±15 years	23 (69.7%) / 10 (30.3%)
Conroy 2005	bromocriptine, pergolide, pramipexole, carbidopa/levodopa, amantadine	Cohort study	919	None	any		174	others included in cohort	745				Ischemic, ICH		moderate stroke mean 65.4±14.8 vs. severe stroke 67.8±14.1 years	F: moderate stroke 50% vs. severe stroke 47.6%
<u>Ergots</u>																
Bochner 1973	hydergine	RCT and post-hoc cross-over study	21 in RCT (15 proceeded to cross-over)	Double blind then single blind	1 mg t.i.d., oral	12 weeks	11	Placebo	10	geriatric	"convalescing"		"Stroke"		mean 64.6 vs. 63.3 years	15 (71.4%) / 6 (28.6%)
<u>Gamma-aminobutyric acid (GABA) agonists</u>																
Liu 2018	clomethiazole, diazepam	SR/MA	3838 (5 RCTs:	Double blind	any, oral or i.v.	any		Placebo		any	<12 hours		Ischemic, ICH			

Study	Treatment	Study design	Study N	Blinding	Dose, frequency, route	Treatment duration	Treatment N	Comparator	Comparator N	Age criteria	Treatment window	Stroke severity, impairments	Stroke subtype	Other key criteria	Age of included	Sex (M / F)
			clomethiazole 4, diazepam 1)													
<u>Methylxanthines</u>																
Bath 2004a	aminophylline	SR/MA	119 (2 RCTs)	Double Blind	any, i.v. or oral	any		Placebo or not treated			<1 week		Ischemic		mean 74 years	63 of 125 (50.4%) / 62 of 125 (49.6%)
Bath 2004b	pentoxifylline, propentofylline	SR/MA	793 (5 trials: pentoxifylline 4, propentofylline 1)	Double-blind, single blind	any, i.v. or oral	any		Placebo or not treated		any	<7 days		Ischemic	definite or presumed	mean 68 years	491 of 788 (62.3%) / 297 of 788 (37.7%)
Modrau 2020	theophylline + thrombolysis	RCT	64	Double Blind	220 mg, <30 min after start of thrombolytic, i.v.	once	33	Placebo + thrombolysis	31	≥18 years	<5 hours	NIHSS ≥4	Ischemic	hemispheric, receiving thrombolytic therapy within 4.5 hours	median 68 (IQR 57 to 76) vs. 71 (60 to 80) years	39 (60.9%) / 25 (39.1%)
<u>Monoamine oxidase (MAO) inhibitors</u>																
Laska 2005	moclobemide	RCT	89	Double Blind	titrated up to 300 mg b.i.d., oral	6 months	45	Placebo	44	≥18 years	<3 weeks	aphasia 1.0 to 4.0 on Amsterdam-Nijmegen-Everyday-Language-Test	Ischemic, ICH		mean 75 (range 51–94) vs. 74 (45–87) years	57% vs. 56% / 43% vs. 44%

Study	Treatment	Study design	Study N	Blinding	Dose, frequency, route	Treatment duration	Treatment N	Comparator	Comparator N	Age criteria	Treatment window	Stroke severity, impairments	Stroke subtype	Other key criteria	Age of included	Sex (M / F)
Bartolo 2015	selegiline	RCT	47	Double Blind	10 mg daily, oral	6 weeks	23	Placebo	24		<2 weeks	MMSE 10 to 23; FIM ≤60	Ischemic, ICH		median 67 (IQR 55 to 75) vs. 64.5 (60. to 69.7) year	21 (44.7%) / 26 (55.3%)
Mood stabilizers																
Mohammadianinejad 2014	lithium	RCT	66	Double Blind	300 mg b.i.d., oral	30 days	32	Placebo	34	50 to 80 years	<48 hours	MRC grade 0 to 3 in at least 1 upper limb	Ischemic	MCA territory, not cardioembolic	mean 62.4±5.1 vs. 62.5±5 years	41 (62.1%) / 25 (37.9%)
Neuropeptides																
Ziganshina 2020	Cerebrolysin	SR/MA	1601 (7 RCTs)		any	any		Placebo or not treated		any	<48 hours		Ischemic			
Bornstein 2018	Cerebrolysin	SR/MA	1879 (9 RCTs)	Double blind	30 to 50 ml daily, i.v.	10 to 21 days		Placebo			<72 hours		Ischemic	MCA territory	18 to 88 years	
Chang 2016	Cerebrolysin + rehab	RCT	66	Double Blind	30 ml daily, i.v. + 2h PT, 1h OT	21 days	34	Placebo + 2 hours PT, 1 hour OT	32	18 to 80 years	<7 days	FMA 0 to 84	Ischemic		64.2±11.5 years	53 (80.3%) / 13 (19.7%)
Woo 2020	Cerebrolysin	RCT	50	Double Blind	10 ml q8h, i.v.	14 days	25	Placebo	25	18 to 80 years	<96 hours		SAH	aneurysmal	53±10 (range 34 to 78) years	16 (32%) / 34 (78%)
N-Methyl-D-Aspartate (NMDA) agonists																
Cherry 2014	cycloserine	RCT	20	Double-blind	250 mg, 1h before motor training, oral	once	10	Placebo	10	≥18	≥6 months	Unilateral upper and lower extremity weakness	Ischemic, ICH		Mean 55.3±9.6 vs. 51.4±11.0 years	11 (55%) / 9 (45%)
NMDA antagonists																

Study	Treatment	Study design	Study N	Blinding	Dose, frequency, route	Treatment duration	Treatment N	Comparator	Comparator N	Age criteria	Treatment window	Stroke severity, impairments	Stroke subtype	Other key criteria	Age of included	Sex (M / F)
Beladi Moghadam 2021	memantine	RCT	53	None	20 mg q8h for 5 days then daily, oral	3 months	24	"Not treated"/not given product under investigation	29	≥18 years	≤24 hours	NIHSS <17	Ischemic		mean 75±6.4 vs. 71±5.9 years	22 (41.5%) / 31 (58.5%)
Berthier 2009	memantine + constraint induced aphasia therapy (CIAT)	RCT	28	Double Blind	10 mg b.i.d., oral	16 weeks then + CIAT for 2 weeks then memantine alone for 2 weeks; 24 weeks of open-label	14	Placebo + 2 weeks CIAT; 24 weeks of open label memantine	14	18 to 70 years	≥1 year	aphasia by Western Aphasia Battery (WAB)	Ischemic, ICH		mean 53.7 (SE 2.1) vs. 48.5 (SE 2.1) years	18 (64.3%) / 10 (35.7%)
<u>Norepinephrine / noradrenergics</u>																
Ward 2017	atomoxetine	RCT	12	Double Blind	40 mg, 60 min prior to 2h of motor training for 10 sessions, oral	10 sessions	6	Placebo	6	≥21 years	≥6 months	moderate to severe UE deficit (inability to extend MCP joint ≥10°, wrist 20°)	Ischemic, ICH		mean 55 (range 35 to 66) years	6 (50%) / 6 (50%)
Zittel 2007	reboxetine	RCT cross-over study	10	Double Blind	6 mg prior to therapy session, oral	once	10	Placebo	10		"chronic"	impairment of fine finger movements, paresis of upper extremity (MRC 4)	"Stroke"		mean 65±14 years	7 (70%) / 3 (30%)

Study	Treatment	Study design	Study N	Blinding	Dose, frequency, route	Treatment duration	Treatment N	Comparator	Comparator N	Age criteria	Treatment window	Stroke severity, impairments	Stroke subtype	Other key criteria	Age of included	Sex (M / F)
Opioid antagonists																
Ortiz 2021	naloxone, nalmefene	SR	naloxone 96 (4 studies), nalmefene 916 (3 studies)	Double blind, single blind, open label	any, once, i.v.	once / once; 10 days	64 / 543	None, placebo or "not treated"	32 / 373	>18 years	Naloxone: 8 - 60 h; 12 h; 24 h; 3-24 h / Nalmefene: 6 h; 6 h; 3 days		Ischemic			
Peripheral chemoreceptor agonists																
Li 2004	almitrine-raubasine	RCT	74	Double Blind	30 mg/10 mg b.i.d., oral	3 months	38	Placebo	36	40 to 80 years	1 to 3 months		Ischemic	MCA territory	mean 61.9±10.5 vs. 62.6±10.5 years	53 (71.6) / 21 (28.4%)
Potassium channel blockers																
Page 2020	Dalfampridine	RCT	377	Double Blind	7.5 mg b.i.d., 10 mg b.i.d., oral	12 weeks	126, 125	Placebo	126	≥18 years	≥6 months	mRS 1 to 3, stable walking deficit	Ischemic		mean 62.9±10.77 years	241 (63.9%) / 136 (36.1%)
Pyrazolones																
Fidalgo 2022	edaravone	SR/MA	50536 (14 observational, 5 RCTs)		60 mg (14 studies), other (5 studies) daily	7 days (2); 14 days (12); unclear (5)	18137	Placebo, not treated	32399	>18 years			Ischemic			
Hu 2021	edaravone + i.v. tPA	SR/MA	1877 (17 RCTs)		60 mg daily	7 days (5); 10 days (1); 14 days (10); 30 days (1)	939	i.v. rtPA alone	938				Ischemic	treated with i.v. rt-PA		

Study	Treatment	Study design	Study N	Blinding	Dose, frequency, route	Treatment duration	Treatment N	Comparator	Comparator N	Age criteria	Treatment window	Stroke severity, impairments	Stroke subtype	Other key criteria	Age of included	Sex (M / F)
Xu 2021	edaravone dexborneol	RCT	1194	Double-Blind	edaravone 30 mg + dexborneol 7.5 mg q12h, i.v.	14 days	599	edaravone alone	595	35 to 80 years	<48 hours	NIHSS 4 to 24, NIHSS motor score ≥2	Ischemic		median 62.96 (IQR 55.38, 68.96) vs. 62.86 (55.72, 70.12) years	M: 811 (67.9%)
Qin 2022	edaravone	SR/MA	3454 (38 RCTs)		30 to 60 mg daily	14 to 30 days	1734	Placebo (1 RCT), usual care (rest)	1720		<7 days		ICH		means 45.8 to 71.03 years	
Racetams																
Ricci 2012	Piracetam	SR/MA	1002 (3 RCTs)		any, i.v. or oral			Placebo or open control		any	<3 days		Ischemic	exclude posterior circulation strokes, GCS <4, mass effect midline shift on early scan	40 to 85 years	
Zhang 2016	Piracetam	SR/MA	261 (7 RCTs)		any			Placebo				Aphasia	"stroke"		mean from 50.9 to 66.6 years	
Vasodilators																
Wu 2015	buflomedil	SR/MA	2756 (26 RCTs)		100 mg to 600 mg, i.v. in 24 studies	10 days to 3 weeks		Placebo or usual care		any	"acute stroke" (within first few days of onset)		Ischemic		means 58 to 75 years	M: 45% to 80%

Study	Treatment	Study design	Study N	Blinding	Dose, frequency, route	Treatment duration	Treatment N	Comparator	Comparator N	Age criteria	Treatment window	Stroke severity, impairments	Stroke subtype	Other key criteria	Age of included	Sex (M / F)
Ni 2020	cinepazide	RCT	937	Double Blind	320 mg daily, i.v.	14 days	466	Placebo	471	18 to 80 years	<48 hours	NIHSS 7 to 25	Ischemic	anterior cerebral circulation	mean 61.2±10.0 years	621 (66.3%) / 316 (33.7%)
